# Electrodialytic Processes: Market Overview, Membrane Phenomena, Recent Developments and Sustainable Strategies

**DOI:** 10.3390/membranes10090221

**Published:** 2020-09-02

**Authors:** Laurent Bazinet, Thibaud R. Geoffroy

**Affiliations:** Department of Food Sciences, Laboratoire de Transformation Alimentaire et Procédés ÉlectroMembranaires (LTAPEM, Laboratory of Food Processing and Electromembrane Processes), Institute of Nutrition and Functional Foods (INAF), Dairy Research Center (STELA), Université Laval, Quebec, QC G1V0A6, Canada; thibaud.geoffroy.1@ulaval.ca

**Keywords:** electrodialysis, ion-exchange membranes, desalination, wastewater remediation, food coproduct valorization, fouling, salinity gradient power, electroconvection, pulsed electric field, eco-efficiency, sustainable development

## Abstract

In the context of preserving and improving human health, electrodialytic processes are very promising perspectives. Indeed, they allow the treatment of water, preservation of food products, production of bioactive compounds, extraction of organic acids, and recovery of energy from natural and wastewaters without major environmental impact. Hence, the aim of the present review is to give a global portrait of the most recent developments in electrodialytic membrane phenomena and their uses in sustainable strategies. It has appeared that new knowledge on pulsed electric fields, electroconvective vortices, overlimiting conditions and reversal modes as well as recent demonstrations of their applications are currently boosting the interest for electrodialytic processes. However, the hurdles are still high when dealing with scale-ups and real-life conditions. Furthermore, looking at the recent research trends, potable water and wastewater treatment as well as the production of value-added bioactive products in a circular economy will probably be the main applications to be developed and improved. All these processes, taking into account their principles and specificities, can be used for specific eco-efficient applications. However, to prove the sustainability of such process strategies, more life cycle assessments will be necessary to convince people of the merits of coupling these technologies.

## 1. Introduction

As shown recently by the Covid-19 sanitary crisis, the major global issue of the modern society is the protection of the human health. The quality of the water that people drink, the food they eat and the environment in which they live is the most important factor affecting human health. Indeed, according to the World Health Organization, 29% of the global population (2.2 billion people) globally lack access to safe water at home [1] while waterborne and foodborne diarrheal diseases are responsible for the death of an estimated two-million people annually, mostly children [2]. Furthermore, from longstanding to emerging hazards, environmental factors are a root cause of a significant burden of death, disease and disability, and the resulting impacts are estimated to cause about 25% of death and disease worldwide [3].

**Figure 1:**
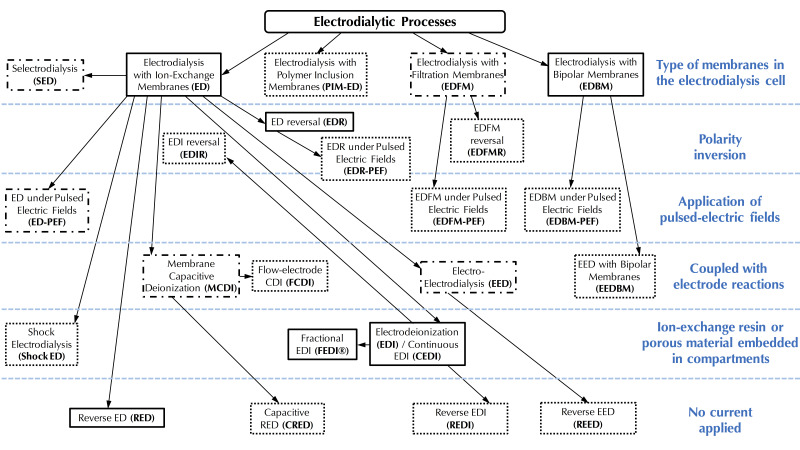
Electrodialytic processes and their characteristics. In solid lines (**^_____^**), technologies available at an industrial scale, in dotted lines (**- - - - -**), technologies at laboratory scale, combination of solid and dotted lines (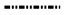), technologies for which scale-up is underway.

In this context of preserving and improving human health, electrodialytic processes are very promising perspectives. Indeed, these processes allow the treatment of water, preservation of food products, production of bioactive compounds having health benefits (antihypertensive, antidiabetic, hypocholesterolemic, antimicrobial, etc.), extraction of organic acids, and recovery of energy from natural and waste waters with minimal environmental impact [4,5,6]. The mother technology of electrodialytic processes is electrodialysis (ED), whose principle is based on the application of an electric potential as a driving force to transport charged species through permselective ion-exchange membranes (IEM). Ions migrate from the diluate (the solution to be demineralized) to the concentrate (the solution recovering the ions): cations cross the cation-exchange membranes (CEM) and anions cross the anion-exchange membranes (AEM), respectively. The overall process balance leads to the separation and concentration of ions in the concentrate as well as the purification by demineralization of the feed stream (diluate), usually water [4]. The ED process is economically competitive with other processes, such as ion-exchange (IX) or nanofiltration (NF), for demineralization rates up to 70% regardless of the mode of operation (batch or continuous). Indeed, the operating cost doubles as the demineralization level increases from 50% to 70%, and then, to 90% [7]. Many electrodialytic processes are derived from the principle of ED and the use of such equipment (Figure 1). The main derived processes commercially available at an industrial scale are electrodialysis with bipolar membranes (referred to as either EDBM or BMED) for which bipolar membranes, dissociating water molecules under the effect of current, are stacked alternatively with IEMs, electrodialysis reversal (EDR) when a polarity inversion is applied during the process to decrease fouling, electrodeionization (EDI) or continuous electrodeionization (CEDI) when some compartments are filled with beads of ion-exchange resin to improve the conductivity of the cell, membrane capacitive deionization (MCDI) a combination of ion-exchange resin beads filling and electrode reaction to produce pure water (Figure 1). Taking into account their principles and specificities, all these processes can be used or adapted to special eco-efficient applications for the sake of improving human health and the well-being of populations.

The aim of the present review is not to scrutinize all the theoretical aspects associated to equations governing membrane migration, mass transfer and related phenomena, already presented by many authors [4,8,9,10,11], but rather to give a global portrait of the most recent developments in electrodialytic membrane phenomena and their uses or potential uses in sustainable strategies or in a circular economy. For this purpose, the authors mainly focused on articles reported in the literature from 2015 to June 2020. The review starts with an overview of the electrodialytic system market, including the global revenues, the main system manufacturers and their evolution in terms of number during the last 50 years. Then, the principles and state of the art on membrane phenomena, limiting current density, electroconvective vortices and pulsed electric field are described and the main equations associated to mass transfer are shortly presented. After that, the most recent developments based on electrodialytic membrane phenomena such as applications of electroconvective vortices, use of pulsed-electric field, electrodeionization and shock electrodialysis are discussed. Based on these new developments or technologies, the integration of electrodialytic technologies in sustainable strategies or use of eco-efficient new electrodialytic technologies such as electrodialysis with filtration membrane are presented and proposed.

## 2. Overview of Electrodialytic Equipment Market and Manufacturers

### 2.1. Electrodialytic Equipment Market

**Figure 2:**
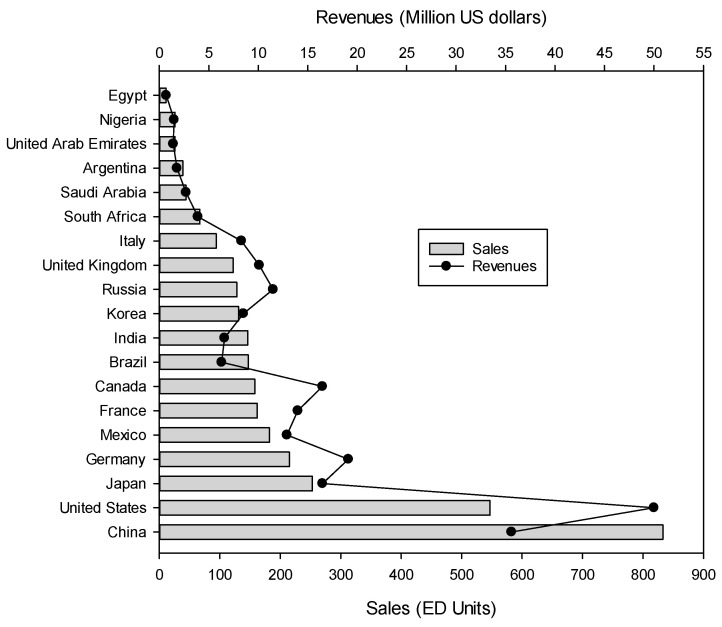
Forecast sales (in number of conventional electrodialysis Units) and revenues (in million US dollars) by countries for 2020.

Among other technologies, our electrodialytic equipment market overview focuses on conventional electrodialysis (ED), electrodialysis reversal (EDR), electrodeionization (EDI)/continuous electrodeionization (CEDI) and electrodialysis with bipolar membranes (EDBM), since they are the most widespread technologies amongst electrodialytic systems. Hence, the worldwide market for electrodialysis systems was of 318.4 million USD in 2019 and is expected to reach 458 million USD by the end of 2025 with roughly a 5.5–5.8% annual growth rate over the next five years [12,13]. China is the country buying the highest number of electrodialysis units in the World with forecast sales of 833 units in 2020 just in front of the USA with 547 units (Figure 2) [13]. ED equipments are sold in many countries around the World, but the main markets for such an equipment are concentrated in 8 countries (China, USA, Japan, Germany, Mexico, France, Canada and Brazil) representing more than 75% of all ED unit sales. In terms of revenues, the order between China and the USA is reversed, since the USA has the highest revenues, close to 50 million USD. In terms of revenues, the USA, China, Japan and Germany represent more than 53% of the total world revenues [13].

Electrodialytic systems are used all around the world for five main purposes: (1) water desalination such as desalination of sea water, desalination of well water, denitrification, treatment of brackish water, etc., (2) waste water treatment such as desalination and recycling of drainage from industrial processes, desalination of leachate from landfill site, desalination and recycling of drainage from activated sludge, etc., (3) food applications for removal of tartaric acid from wine, desalination of cheese whey, purification of lactose, lactulose, and galactose, desalination of molasses, etc., (4) biotechnology/pharmaceutical applications such as desalination of amino acid solution, refining of amino acids, desalination of pharmaceutical intermediates, recovery of blood plasma proteins, acid and base production, solvent purification, etc. and (5) others such as rinse water for electronics processing, reuse of electroless plating baths, treatment of boiler feed water, production of ultrapure water.

### 2.2. Electrodialytic Equipment Manufacturers

In 2020, more than 45 companies are manufacturing and selling electrodialytic equipments around the world. Amongst them, 70.5% are manufacturing ED/EDR equipments, 47.7% EDI/CEDI/MCDI/FEDI equipments and only 18.2% EDBM equipments. More precisely, 65.9% are selling just one type of equipment, 31.8% two types and 2.3% the three types. Generally speaking, the EDI or ED/EDR are coupled with reverse osmosis units when purified water or pure water is sought.

The key companies of electrodialytic equipments, based on 2019 revenues, are by alphabetical order: AGC Engineering (Tokyo, Japan, formely Asahi Glass), Astom (Tokyo, Japan, fusion of Tokuyama Corporation and Asahi Kasei Corporation in 1995), C-Tech Innovation Ltd. (Capenhurst, United Kingdom), Doromil (Beijing, China), Electrosynthesis Company (Lancaster, NY, USA), Eurodia (Pertuis, France), Evoqua Water Technologies LLC. (Pittsburgh, PA, USA), Fumatech BWT GmbH (Bietigheim-Bissingen, Germany), Hangzhou Iontech Environmental Technology Co., Ltd. (Hangzhou, China), LTD Innovative Enterprise Shchekinoazot (Shchekino, Russia), Mega A.S. (Stráž pod Ralskem, Czech Republic), PCCell GmbH (Heusweiler, Germany), Saltworks Technologies Inc. (Richmond, BC, Canada), Shandong Tianwei Membrane Technology Co., Ltd. (Weifang, China), SUEZ–Water Technologies & Solutions (Trevose, PA, USA), SnowPure Water Technologies (San Clemente, CA, USA). Unlike in many market reports, WGM Sistemas was not included here as an ED unit manufacturer, since it has a partnership with the French company Eurodia for ED units, and is mainly a sales representative of this company. Some companies are spread in all continents such as Evoqua Water Technologies LLC., SUEZ–Water Technologies & Solutions or Eurodia. For example, Evoqua has more than forty offices around the world. Amongst the companies, SUEZ–Water Technologies and Solutions, the global greatest company in the electrodialytic system industry, accounted for about 15% of the revenue market share in 2019, followed by Evoqua Water Technologies LLC (11%) and then by a close group formed by PCCell GmbH, Eurodia and AGC Engineering (between 7% and 9%). The top ten manufacturers account for more than 65% of the revenue market [12].

**Figure 3:**
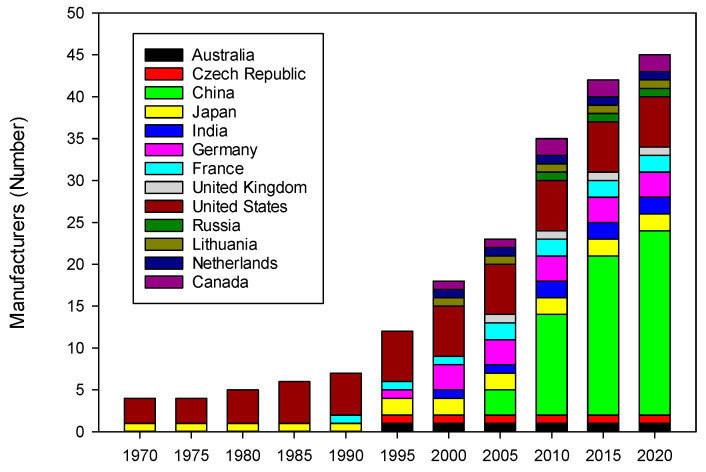
Evolution of the number of electrodialytic system manufacturers around the World from 1970 to 2020.

Having a look at the evolution of the number of electrodialytic unit manufacturers around the World in the last 50 years, it appears that in the 1970s–1980s, only 4–6 companies, from the USA and Japan, shared the market of electrodialytic systems mainly composed of conventional electrodialysis (Figure 3). The pioneer company, and the leader in electrodialysis during many years was Ionics, acquired in 2005 by GE Water and Process Technologies, itself acquired by SUEZ in 2017 to form SUEZ–Water Technologies and Solutions. Indeed, the first ED desalination plant was built by Ionics in 1954 for Aramco (Saudi Arabia) [10]. A few years later, ED units for brackish water desalination were built in South Africa [14]. In 1988, Eurodia was created and participated to the dissemination of conventional electrodialysis technology as well as EDR and EDBM. Then, from 1990 to 2005, companies from other countries (Australia, China, Czech Republic, India, Germany, United Kingdom, Lithuania and Netherlands) joined the ranks of the electrodialytic system manufacturers. During this period, the emergence of bipolar membranes, leading to new and different applications where pH differences play a major role (production of acids and bases, recovery of organic acids), the growing importance of resource recovery and related selective recovery of anions and metals, and the design of alternative electrodialysis stack configurations, which are intended for selective separation in a series of applications [4], in parallel with an increase in population and urbanization of the Asia-Pacific region accelerated the needs for electrodialytic systems. As an example, China experienced a very significant boom in its urbanization from the 1990s with the rise of the Chinese economy [15,16,17]. Hence, between the beginnings of the country’s opening to a market economy in 1978 and 2013, the Chinese urban population increased more than fourfold, from 172 to 731 million people. As a consequence, in the last fifteen years and up to now, the new manufacturers were mainly coming from China. This second, quite exponential, increase is probably related to the acceleration of the Asia–Pacific population urbanization, to support the spectacular growth of the economy in this region: In China, the urban population exceeded the 50% threshold for the first time in 2011. Such a growing urban population is presaged to cause a surge in China’s and more generally Asian’s consuming capacity in the next decades [18]. This also means more potable water to be produced and more waste water generated by the industries to be treated in order to avoid water spoilage and decrease environmental impacts. This also explains the fact, that these new companies in electrodialytic systems, when looking at their activities, are orientated to the production of potable water and water treatment. In 2020, seawater desalination and recycling wastewater represents more than 70% of the electrodialytic applications. Considering the future trends in electrodialytic system applications, water will be, for the next decades, a main concern and a still increasing market for the application and development of electrodialytic systems.

## 3. Membrane Phenomena

During ED process, different phenomena appear following mass transfer such as concentration gradients at the membrane interfaces, concentration polarization of the membrane and reaching a limiting current density after the transport, more or less forced, of ions across the ion-exchange membranes. Furthermore, the formation of electroconvective vortices at the surface of the membranes has recently been reported and visualized when the limiting current value is exceeded, increasing so the mass transfer.

### 3.1. Mass Transfer

**Figure 4:**
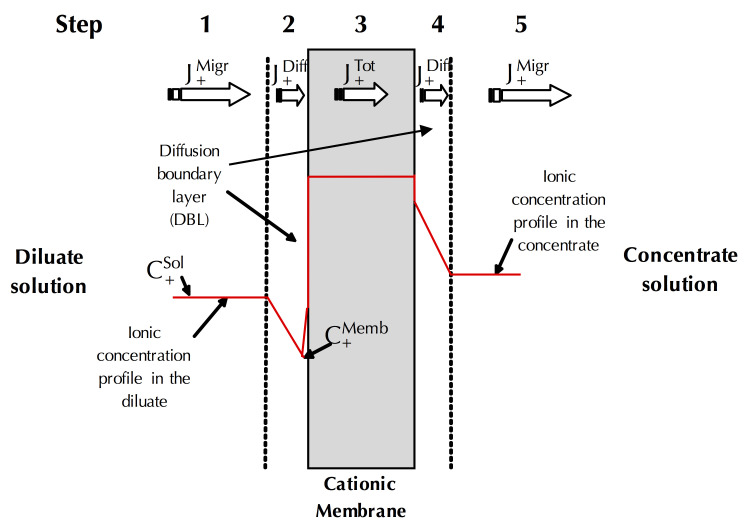
Schematic ion concentration profiles in the diffusion boundary layers (DBL) close to a cation-exchange membrane under an electric field, under the limiting current density, and the different mass transfer mechanisms involved in cation migration (Adapted from Bazinet and Castaigne [11]).

During conventional ED, the mass transfer from a diluate solution to the concentrate solution through the ion-exchange membrane occurs in five steps due to the formation of diffusion boundary layers (DBL) of laminar flow on both side of the membrane (Figure 4) [11]:

1. The *transport by migration* ($J_{i}^{Migr}$) of charged species under an electric field from the diluate solution to the DBL near the membrane according to the Faraday’s law:

(1) $$J_{i}^{Migr}=-D_{i}\frac{F}{R_{U}T}z_{i}C_{i}^{Sol}\left(\frac{\Delta U}{\Delta X}\right)$$

with $J_{i}^{Migr}$ the flux by electromigration of the species i (in mole/s.m^2^), D_i_ the diffusion coefficient of the species i (in m^2^/s), F the Faraday constant (96,500 C/mol), z_i_ the valence of the species i, $C_{i}^{Sol}$ the concentration of the species i in the solution (in mole/m^3^), ∆U the potential difference applied at the electrodes (in V), R_U_ the ideal gas constant (8314 J/mol.K), T the temperature of the solution (in K) and ∆X the distance between the electrodes (in m).

2. The *transport by diffusion* ($J_{i}^{Diff}$) through the partially desalinated boundary layer of the membrane given by the Fick’s first law:

(2) $$J_{i}^{Diff}=\frac{-D_{i}(C_{i}^{Sol}-C_{i}^{Memb})}{\delta }$$

with $J_{i}^{Diff}$ the flux by diffusion of the species i (in mole/s.m^2^), $C_{i}^{Memb}$ the concentration of the species i in the diffusion boundary layer at the interface of the membrane on the diluate side (in mole/m^3^) and δ the thickness of the boundary layer (in m).

3. The *transport through the membrane* ($J_{i}^{Tot}$) given by the Nernst–Planck’s equation:

(3) $$J_{i}^{Tot}=-D_{i}\left(\frac{\Delta C_{i}}{\Delta X}-z_{i}\frac{F}{R_{U}T}C_{i}\frac{\Delta U}{\Delta X}\right)+C_{i}\nu$$

with $J_{i}^{Tot}$ the flux of the species i through the membrane (in mole/m^2^·s), C_i_ the concentration of the species i (in mol/m^3^) and ν the convection velocity (in m/s).

Although the Nernst–Planck’s equation did not take into account the coupling of the fluxes between species migrating through the membrane, this equation describes major factors having an effect on the transfer of ions. This theory is based on the assumption that an ion-exchange membrane is considered as a thick phase equivalent to a solution, separating two adjacent aqueous phases. To describe correctly the transport of charged species through the membranes, it is necessary to couple the Nernst–Planck equation to the electroneutrality condition:

(4) $$\sum z_{i}\cdot C_{i}=0$$

Or, considering the transport numbers of the anions and cations through the same membrane:

(5) $$\sum \left(Nbt^{-}+Nbt^{+}\right)=1$$

with Nbt^−^ the transport number of the anions and Nbt^+^ the transport number of the cations given by the following equation:

(6) $$Nbt_{i}=\frac{FJ_{i}z_{i}}{d_{I}}$$

With F the Faraday constant (96,500 C/mol), J_i_ the molar flux of the species i (in mole/s·m^2^), z_i_ the valence of the species i, $d_{I}=\frac{I}{S}$ the current density (in A/m^2^ of electrode).

4. The *transport by diffusion* ($J_{i}^{Diff}$) through the partially mineralized diffusion boundary layer on the other side of the membrane given, as previously, by the Fick’s first law.
5. And, finally, the *transport by migration* ($J_{i}^{Migr}$), of the charged species from the mineralized membrane diffusion boundary layer to the concentrate solution, given as previously by the Faraday’s law.

### 3.2. Concentration Gradient and Concentration Polarization

#### 3.2.1. Concentration Gradient

**Figure 5:**
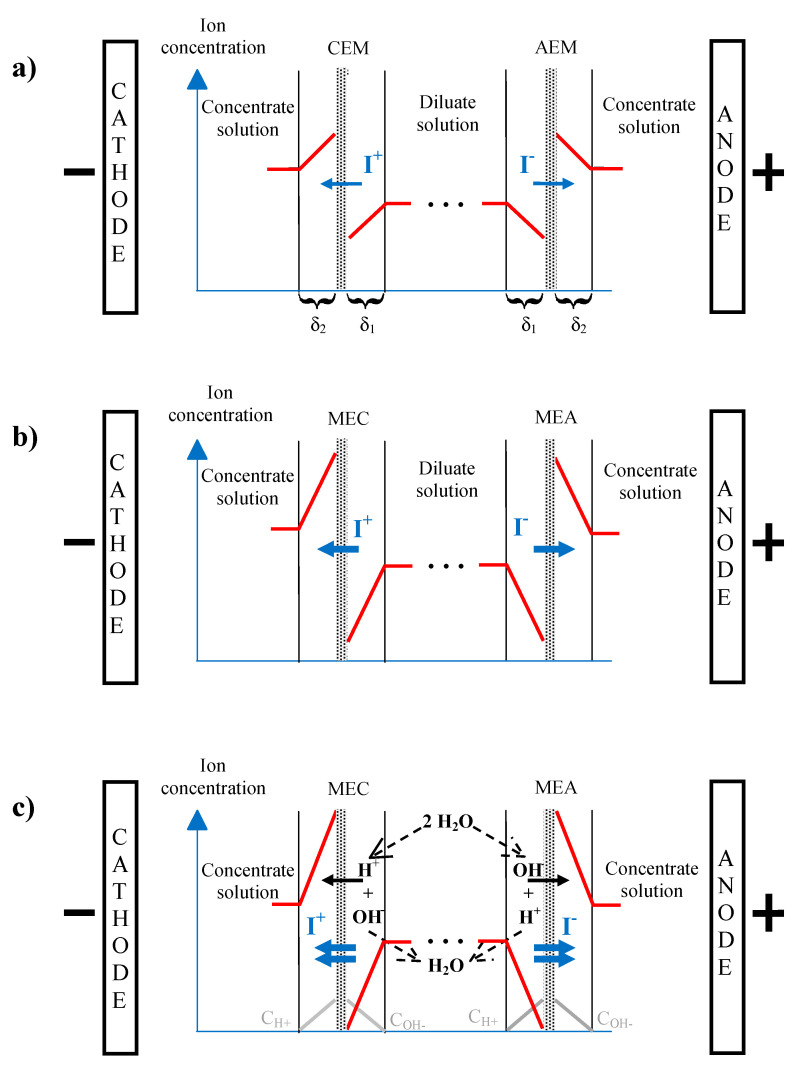
Steps in concentration polarization phenomenon at membrane interface during electrodialysis: (**a**) formation of concentration gradients, (**b**) reaching the limiting current density and, (**c**) overpassing the limiting current density and irreversible water dissociation. CEM: cation-exchange membrane; AEM: anion-exchange membrane; δ_1_: diluate boundary layer; δ_2_: concentrate boundary layer; C_H_^+^: concentration in H^+^ and C_OH_^−^: concentration in OH^−^ (Adapted from Bazinet [22]).

When a current is applied to an electrodialysis cell, a variation in the concentration profile close to the membranes appears, in the DBLs relative to the concentrate and diluate, between the turbulent zones of the solution and the membrane. After a short transitional period, concentration gradients are established (Figure 5a). Two phenomena are combined to establish this non-uniform concentration profile of the solution at the membrane interface [19]:

1. The presence of a laminar flow diffusion boundary layer at the vicinity of the membranes;
2. The difference of ion transport numbers in the solution and in the membrane, which results in a difference between the ionic flux in the solution and in the membrane.

For example, for NaCl in aqueous solution, it was reported that the flux of Na^+^ ion through the cationic membrane $Nbt_{Na^{+}}^{Memb}$ ≈ 1 is higher than the transport of this same ionic species in the solution $Nbt_{Na^{+}}^{Sol}$ ≈ 0.39, while $Nbt_{Cl^{-}}^{Memb}$ is close to zero and $Nbt_{Cl^{-}}^{Sol}$ ≈ 0.61 [20,21]. $Nbt_{Na^{+}}^{Memb}$ is close to 1 since Na^+^ is the only cationic species present, in this case, able to transport the current through the cation-exchange membrane and vice versa for Cl^−^ with the anion-exchange membrane ($Nbt_{Cl^{-}}^{Memb}$ ≈ 1). Furthermore, at steady state, at any point of the solution and even of the ED module, the ion flux is proportional to the transport number.

Since the solution entering the ED cell flows in a turbulent regime, the ionic concentration in the solution can, consequently, be considered as homogeneous. The flux of an ionic species in the solution under the effect of the electric field is then equal to:

(7) $$J_{i}^{Sol}=\frac{Nbt_{i}^{Sol}d_{I}}{F}$$

The flux of the ionic species crossing the membrane as expressed by the Faraday’s law and as a function of its ion transport number is given by the following equation:

(8) $$J_{i}^{Memb}=\frac{Nbt_{i}^{Memb}d_{I}}{F}$$

Since $Nbt_{i}^{Memb}$ > $Nbt_{i}^{Sol}$ this implies that $J_{i}^{Memb}$ > $J_{i}^{Sol}$. This flux difference results in a depletion of the ionic species in the DBL at the diluate-CEM side and by an excess of these ions in the DBL at the concentrate-CEM side. Moreover, this excess of ions at the concentrate-CEM side is intensified by the co-ions excluded by the membrane and that are trapped in the DBL. A similar phenomenon occurs at the interfaces of the anionic membranes. In a general manner, at the CEM-diluate and AEM-diluate interfaces, the respective concentrations in cations and in anions decrease, to impoverish the diluate in salts. At the same time, at the CEM-concentrate and AEM-concentrate interfaces, the anion and cation concentrations increase respectively to enrich the concentrate solution in salts. At steady state, the flux of the ionic species from the diluate solution to the concentrate solution should be constant. There is then another transport mechanism which compensates for the depletion of ionic species. This mechanism is called the diffusion. It occurs due to concentration gradients formed on both sides of the membrane.

Since the flux of the ionic species through the DBL is equal to a fraction of the total quantity of equivalent that can be transported according to the Faraday’s law, this fraction is calculated by the transport number of this ion:

(9) $$J_{i}^{C\lim}=\frac{Nbt_{i}^{C\lim}d_{I}}{F}$$

with $J_{i}^{C\lim}$ the flux of ions by electrotransport (in mole/m^2^·s), $Nbt_{i}^{C\lim}$ the transport number of the ion in the DBL, $d_{I}=\frac{I}{S}$ the current density (in A/m^2^ of electrode) and F the Faraday constant (96,500 C/mol).

The flux of this ionic species by diffusion through the same DBL is expressed, as previously, by the Fick’s first law:

(10) $$J_{i}^{Diff}=-D_{i}\frac{\left(C_{i}^{Sol}-C_{i}^{Memb}\right)}{\delta }$$

At steady state, the ionic flux and the diffusion of ions due to the concentration gradient established between the turbulent solution and the membrane surface, the DBL, are equivalent:

(11) $$J_{i}^{Memb}-J_{i}^{Sol}=J_{i}^{Diff}=-D_{i}\frac{\left(C_{i}^{Sol}-C_{i}^{Memb}\right)}{\delta }$$

or

(12) $$\frac{d_{I}\left(Nbt_{i}^{Memb}-Nbt_{i}^{Sol}\right)}{F}=\frac{-D_{i}(C_{i}^{Sol}-C_{i}^{Memb})}{\delta }$$

This equation is the well-known Nernst’s equation and can also be expressed as:

(13) $$d_{I}=-\frac{FD_{i}(C_{i}^{Sol}-C_{i}^{Memb})}{\left(Nbt_{i}^{Memb}-Nbt_{i}^{Sol}\right)\delta }$$

where $d_{I}$ is the current density (in A/m^2^ of electrode), F the Faraday constant, D_i_ the salt diffusion coefficient, $C_{i}^{Sol}$ the concentration of the species i in the solution (in mole/m^3^), $C_{i}^{Memb}$ the concentration of the species i in the DBL at the interface of the membrane on the diluate side (in mole/m^3^), $Nbt_{i}^{Memb}$ and $Nbt_{i}^{Sol}$ the salt counterion effective transport number in the membrane and solution, respectively, and δ the thickness of the DBL (in m).

Then, if the mass transfer is further increased by increasing the voltage applied between the electrodes of the ED cell, this will result in an increase of the current density and of concentration gradients in the DBLs. The driving force for the diffusion (Figure 5b) is then more important. Effectively, $C_{i}^{Memb}$ at the diluate/CEM side will decrease resulting in an increase of the $\left(C_{i}^{Sol}-C_{i}^{Memb}\right)$ difference in the DBL.

#### 3.2.2. Concentration Polarization

**Figure 6:**
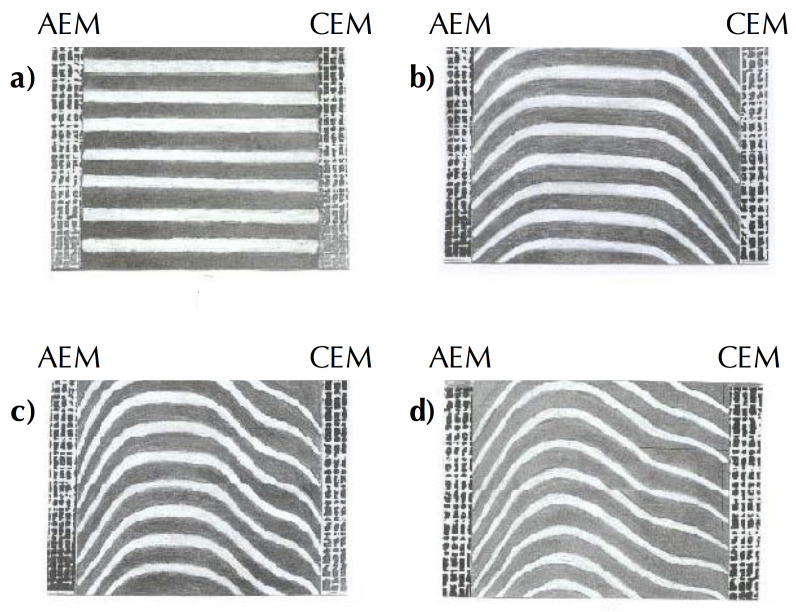
Interferograms of a 1.0 × 10^−2^ mol/L sodium chloride solution in the diluate compartment of an ED cell at different current densities (**a**) 0 A/m^2^, (**b**) 18.5 A/m^2^, (**c**) 59.7 A/m^2^ and (**d**) 126.0 A/m^2^. Experimental conditions: flow rate of 1.26 × 10^−3^ m/s, intermembrane distance of 1.5 × 10^−3^ m, coordinate in the direction of solution feed 1.1 × 10^−2^ m (with permission from Vasil’eva et al. [20]). AEM: Anion-exchange membrane; CEM: Cation-exchange membrane.

Concentration gradients formed on both sides of the membrane result in an accumulation of positive charges on one side and negative charges on the other. This phenomenon is called concentration polarization (CP). The visualization of CP and consequently formation and development of diffusion layers during ED in a wide range of current densities was demonstrated by Vasil’eva et al. [20] (Figure 6).

The polarization effect forces a limiting current density that should not be exceeded. Effectively, if the current is still increased, the limiting concentration of $C_{i}^{Memb}$ ≈ 0 in the CEM-diluate DBL is then reached. At this moment, the resistance of the CEM-diluate DBL highly increases and the current density reaches a limiting value (Figure 5b) [22]. In these conditions, the flux of ions by diffusion reaches a maximum and is given by the following equation:

(14) $$J_{\max}^{Diff}=\frac{D_{i}C}{\delta }$$

when the flux of ions by electrotransport ($J_{i}^{Migr}$) is equal to the flux of ions by maximal diffusion ($J_{\max}^{Diff}$), the ED process is carried out at its optimal mass transfer.

### 3.3. Limiting Current Density (LCD)

#### 3.3.1. Limiting Current Density and Water Dissociation

A subsequent increase in the voltage difference, at the electrodes of the ED stack, will raise the current density but not the mass transfer. The main part of this additional current will not be used for the mass transfer of the ions in the dilute solution toward the concentrate solution but for the dissociation of water molecules [23,24]. Then, water dissociation generates H^+^ and OH^−^ ions, which are immediately taken up by the ion transport (Figure 5c). Generation of H^+^ and OH^−^ ions at the surface of IEMs was first noted by Kressman and Tye [25] and at the surface of bipolar membranes by Frilette [26]. The generation of H^+^ and OH^−^ ions occurs when the electrolyte concentration near the membrane surface reaches values of 10^−3^–10^−4^ mM, and not, as expected, when the concentration of electrolyte reaches zero [27,28,29]. This was revealed by chronopotentiometric measurements [29] and investigations by means of interferometry [27,28].

Furthermore, it is worth emphasizing that when the current density exceeds LCD, the transfer of H^+^ through the CEM is much weaker than transfer of OH^−^ ions through AEM [30]. This was explained by a difference in their transport numbers; ranging from 4 × 10^−5^ to 11 × 10^−2^ for H^+^ and from 3 × 10^−2^ to 6 × 10^−1^ for OH^−^ ions [29,31,32]. In addition, it was also reported that at current values near to the LCD the electrolyte concentration close to the CEM interface is higher than at the vicinity of the AEM. Furthermore, at the vicinity of AEM concentrations of H^+^ and OH^−^ ions are similar to their concentrations in water, while it is not the case for CEM. This was explained by the presence of convection near the surface of the CEM, improving the ion transfer and hampering water dissociation [8,21,30,33]. Another explanation of the preferable water splitting near the AEM is the nature of the membrane ion-exchange groups, which play a crucial role in generation of H^+^ and OH^−^ ions, since water dissociation at the IEM interface occurs via interactions with membrane ion-exchange groups [30,34,35,36]. Hence, Simons showed a significant acceleration of water dissociation reaction by catalysis with ion-exchange groups and that this reaction, with tertiary amino groups, was limited by the reaction stage BH^+^ + H_2_O $\overset{k_{1}}{\underset{k_{-1}}{⇄}}$ B + H_3_O^+^ with a rate constant k_lim_ > 2.5 s^−1^ [35]. This value is five orders higher than the dissociation constant (*k*_d_) without ion-exchange groups. The following classification of membrane ion-exchange groups in ascending order of rate constants was proposed [37]:

−N(CH_3_)_3_^+^
< −SO_3_^−^< −PO_3_H^−^< =NH, −NH_2_< ≡ N< −COO^−^< −PO_3_^2−^k_lim_ (in s^−1^):0
3 × 10^−3^
3 × 10^−2^
10^−1^
1
10
10^2^

According to this classification, using CEM with sulfo groups and AEM with tertiary amino groups the water splitting rate will be 3-orders higher on AEM. Furthermore, acceleration of H^+^ and OH^−^ generation was observed in ED systems containing inorganic ions and hydroxides or organic substances, which are able to precipitate on ion-exchange groups [34,38,39,40].

In addition, in works devoted to the study of CP phenomena it was also reported that near the LCD an increase in system resistance appeared in parallel with the generation of H^+^ and OH^−^ ions [25,34,41]. Both effects have major consequences on the global efficiency of the ED process: increase in system electrical resistance leads to an increase in energy consumption and cost of ED while the generation of H^+^ and OH^−^ to a decrease in current efficiency and appearance of sedimentation when the solution contains pH sensitive components [42,43,44,45,46].

In practice, between 70% and 80% of the limiting current density of the membrane/electrolyte system should not be exceeded in order to prevent reaching this limiting current density at any place of the electrodialysis cell during the whole process.

#### 3.3.2. Determination of the Limiting Current Density

**Figure 7:**
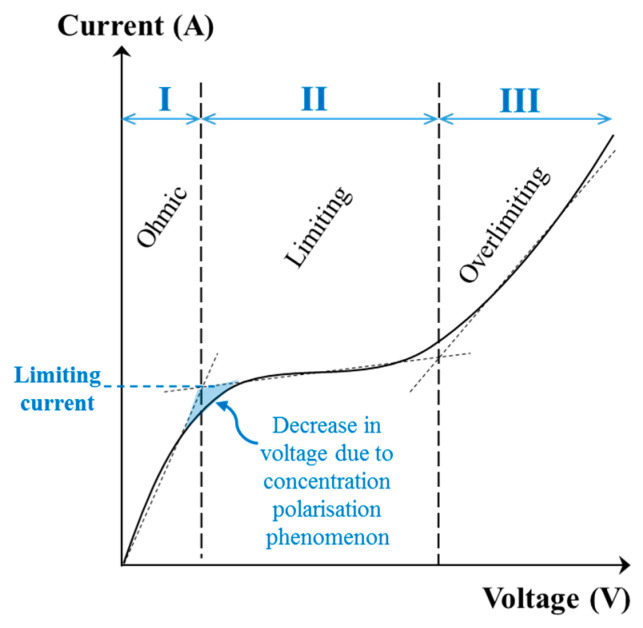
Typical current-voltage curve for an ion-exchange membrane and value of the limiting current (adapted from Bazinet and Castaigne [11]).

The limiting current density can be determined experimentally by drawing a current-voltage curve (Figure 7). The limiting current density (A/m^2^) will be calculated by dividing the value of limiting current (I_lim_) obtained from the curve by the electrode effective surface (en m^2^). The curve obtained shows three distinct regions [10,47]:

1. The ohmic region (**I**) where the current (as well as the current density) increases linearly as a function of the voltage applied. In this region, the system follows the Ohm’s law (U = RI) and the global resistance of the electrodialysis system (R) is fairly constant.
2. A «plateau» region (**II**), named limiting region, where the current remains relatively constant while the voltage increases. This particular value refers to the limiting current density which corresponds to the maximal current value (I_lim_) from which water dissociation begins.
3. A third region (**III**), named overlimiting region, where the current increases when voltage applied is further increased. This region corresponds to an overpassing of the limiting current density during which the electrical energy is used to dissociate water molecules without taking part in solutes separation. This regime is also characterized by the occurrence of exaltation and current-induced convection phenomena (see Section 3.4).

**Figure 8:**
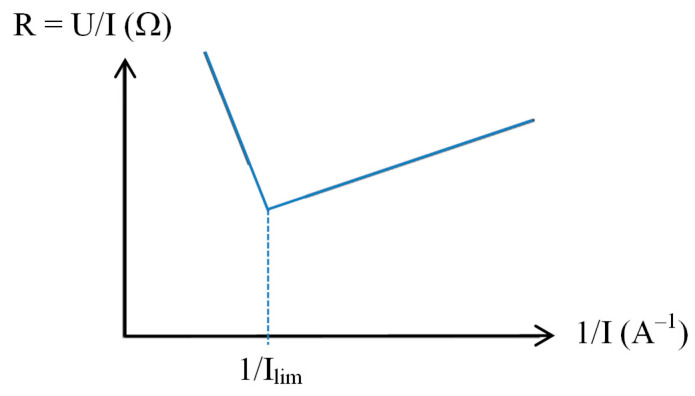
Determination of the reciprocal limiting current value by the method of Cowan and Brown [41].

Another method is frequently used to determine experimentally the limiting current value. A graph of the resistance (U/I) is plotted as a function of the reciprocal current value (1/I) (Figure 8). The reciprocal limiting current value is then at the intersection of the two lines [41].

#### 3.3.3. Calculation of the Limiting Current Density

The limiting current density (in A/m^2^ of electrode) can be approximated by the Lévêque equation [48]:

(15) dIlim=1.47FDiCinlet(NbtiMemb−NbtiSol)h(h2vLDi)13

where F is the Faraday constant, D_i_ the salt diffusion coefficient, C_inlet_ the inlet concentration (in eq./m^3^), h the distance between the membranes (in m), v the average linear solution velocity (in m/s), $Nbt_{i}^{Memb}$ and $Nbt_{i}^{Sol}$ the salt counterion effective transport number in the membrane and solution, respectively, and L the length of the membrane active area (in m).

It is also possible to calculate the limiting current density value by substituting $C_{i}^{Memb}$ by 0 in Equation (13):

(16) $$d_{I_{\lim}}=-\frac{FD_{i}C_{i}^{Sol}}{\left(Nbt_{i}^{Memb}-Nbt_{i}^{C\lim}\right)\delta }$$

It appeared that the limiting current density value is directly proportional to the concentration of charged species in solution. If we want to calculate the limiting current value $d_{I_{\lim}}$, the previous equation is of little interest, because in practice, it is very difficult to measure experimentally the thickness δ of the DBL [49]. Therefore, a similar approach to the one employed in ultrafiltration can be used to measure this limiting current value. The term $\frac{D}{\delta }$ is considered as a mass transfer coefficient, K_m_, which can be related to other physical basic units by Sherwood’s correlations:

(17) NSh=KmdeDi=ANReaNSc0,33

With N_Sh_ the Sherwood number (dimensionless), K_m_ the mass transfer coefficient (in m/s), d_e_ the spacer thickness (in m), D_i_ the diffusivity coefficient (in m^2^/s), A a constant, N_Re_ the Reynolds number (NRe=ρvdeη), a another constant, N_Sc_ the Schmid number ($N_{Sc}=\frac{\eta }{\rho .D}$), ρ the fluid density (in kg/m^3^), v the fluid velocity in the spacer (in m/s), and η the fluid viscosity (in Pa.s or decapoise).

Constants A and a can be determined experimentally and can subsequently be used for the design of similar equipment. From the previous equation, it appears that the mass transfer coefficient K_m_ is a function of the apparatus design (d_e_), the flow type (N_Re_) and the physical properties of the solution (D, ρ and η). The K_m_ value will also vary according to the fluid velocity in the spacer:

K_m_ ≈ v^a^ (18)

For thin spacers, the exponent a varies, in most cases, from 0.5 (for a laminar flow) to 0.90 (for a turbulent flow). In practice, spacers used in ED are very thin (0.2–2 mm), which will contribute to the establishment of a laminar flow, and minimize the electrical resistance of the solution.

The current density applied for conventional electrodialysis in its main industrial applications is generally in the range of 100 to 400 A/m^2^, however in water treatment the current density is <80 A/m^2^. For EDBM at an industrial scale, the current density applied is more between 500 and 1000 A/m^2^.

### 3.4. Overpassing the Limiting Current Density and Occurence of Vortex

The operation of electrodialytic equipments in overlimiting current densities was demonstrated to be responsible for an additional supply of counter-ions at the membrane surface, which occurs because of the exaltation effect and current-induced convection, accompanied by water splitting at the membrane/solution interface [50,51,52]. These phenomena, observed in overlimiting conditions, have major impacts on electrodialytic process performances.

#### 3.4.1. Exaltation Effect

**Figure 9:**
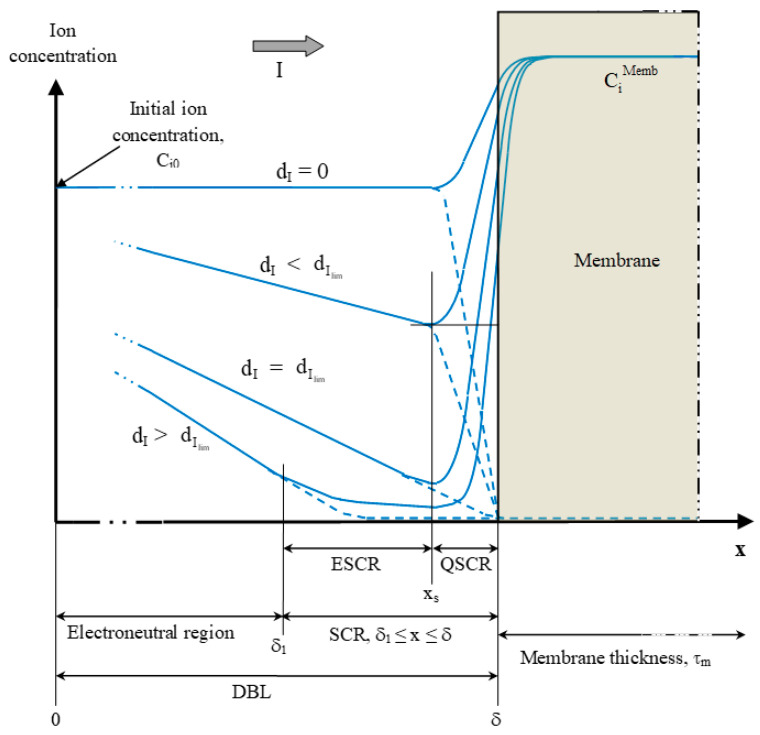
Schematic concentration profiles of counter-ions (**solid line**) and co-ions (**double dashed line**) in the double boundary layer (DBL, thickness δ) as a function of the current density (d_I_) applied. SCR: Space charge region; ESCR: extended space charge region (non-equilibrium part); QSCR: quasi-equilibrium part of SCR (Adapted from Nikonenko et al. [21,51]).

One mechanism of ion transfer when CP is developed is current exaltation. This effect was firstly described, in membrane systems, by Kharkats [53]. Due to CP phenomenon, the increase in current density causes the increase in ion depletion at the interface of the membrane in the DBL of thickness d (Figure 5 and Figure 9), as well as in the space-charge region (SCR) [54] (Figure 9). Indeed, the DBL, at the interface of the membrane, is generally divided into two regions: an electroneutral region (0 ≤ *x* ≤ δ_1_) and the space-charge region (SCR) (δ_1_ ≤ *x* ≤ δ). When in ohmic and limiting conditions, the SCR occupies a negligible space (<10 nm) (Figure 9). However, when overlimiting conditions are reached, the SCR thickness increases due to the appearance of a non-equilibrium zone and then extends up to several hundred nanometers, which is wider than the electric double layer (EDL) thickness (several nanometers) [51]. In this extended SCR, the concentration in counter-ions, although low, is significantly higher than the ones of co-ions (negligible) explaining the fact that this extended SCR does not respect the principle of electroneutrality [54] (Figure 9). In this extended SCR, depleted in ions, exaltation effect arises due to the attraction of counter-ions by-products of water splitting. Indeed, the appearance of OH^−^ and H^+^ ions disturbs the electric field and favors the attraction of counter-ions of the solution towards the membrane interface (exaltation effect) [21]. Hence, the dissociation of water molecules at the membrane surface promotes the effect of exaltation. For example, in desalting channel of ED system with desalination of NaCl solution, the increase of current in the overlimiting region may be due to attraction of Na^+^ by OH^−^ produced on the CEM surface and due to attraction of Cl^−^ by H^+^ produced on AEM surface. The exalted current is described by the mechanism called the Kharkats current. More precisely, in the case of a CEM, while the H^+^ generated at its interface would be used for the transport of the current in the membrane, OH^−^ ions would attract counter-ions such as Na^+^, K^+^, Ca^2+^ and Mg^2+^ towards the interface of the membrane. However, the contribution of the exaltation effect on the increase in ion transfer in overlimiting regime is very low compared to the effect provided by current-induced convection. Indeed, the ratio of diffusion coefficients of counter-ions to diffusion coefficients of water splitting products is of the order of 10^−1^ and this fact leads to the conclusion that the increment in salt counter-ion flux due to the exaltation effect is rather low (around 20%) [21].

#### 3.4.2. Current-induced convection

The most powerful mechanism leading to the essential exceeding of the current above its limiting value is current-induced convection [8,9,21,30,55,56,57]. Two current-induced convection mechanisms, causing the mixing of the depletion region with well conductive bulk, are recognized, namely gravitational convection (minor effect) and electroconvection (major effect).

##### Gravitational Convection

Gravitational convection is caused by natural convection that occurs due to a difference in concentration and/or temperature (larger heat dissipation in the depletion layer) gradient within the same solution. Indeed, the gradients created by these differences generate density gradients. For example, at the membrane surface, the very low conductivity of the extended SCR causes high electrical resistance and promotes a rise in temperature by the Joule effect [57]: the conductivity in this location is 100 to 1000 times lower than in membranes and in the solution at the center of the compartment [58]. In the case of vertically placed membranes, when a density gradient appears due to this temperature rise, the solution at the membrane interface, less dense, tends to go up while under the effect of the earth’s gravitational force, the raw solution, in the center of the compartment, more dense, move towards the zone of the least dense solution and goes down, thus creating a convection. This type of convection can be diminished by positioning the membrane horizontally [59].

This convection event remains negligible for infinitely diluted solutions, since the density differences are almost zero [21]. It has been found that gravitational convection occurs when the Rayleigh number (N_Ra_), product of Grashof and Schmidt numbers, was around 1700 [60]. In addition, experimental data and modeling calculations demonstrate that gravitational convection can have a significant contribution on mass transfer only when the concentration of the solution is relatively high (˃0.05 M) and that the velocity of the solution is low (˂0.4 cm/s) [61,62,63].

##### Electroconvection

**Figure 10:**
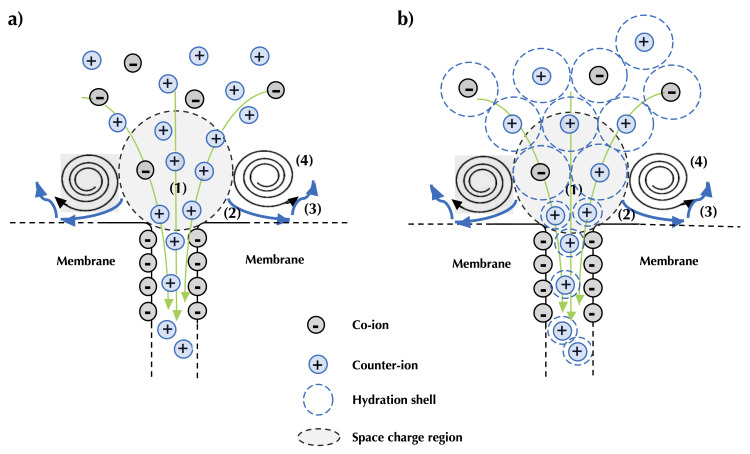
Occurrence of electroconvective vortices at membrane interface due to electroosmosis of the second kind (**a**) as currently accepted (adapted from Nikonenko et al. [21]) and (**b**) hypothetic, taking into account the ion hydration shell.

The other type of convection, referred as electroconvection, originates from the interaction of an electric field and a spatial charge. Electroconvection is the major phenomena responsible for ion transport when the system operates in overlimiting current regime, since it allows a better availability of ions at the membrane interface by supplying fresh solution to the membrane surface and by removing the depleted solution [64,65,66]. Electroconvection can occur in the volume (volumetric electroconvection) and near the membrane surface (electro-osmotic motion). However, due to the close values of diffusion coefficients of cations and anions, volumetric electroconvection does not play noticeable role in the mass transfer [67,68]. Hence, electroconvection near the membrane interface is conventionally considered as the main electroconvection phenomenon contributing to the mass transfer improvement. It is considered as an electro-osmotic motion and may be distinguished into two principal kinds [21,57,69]. Electro-osmosis of the first kind arises under the limiting current region due to the action of tangential electric field upon the quasi-equilibrium diffusion part of DBL (Figure 9), which has almost the same structure to that for zero current; it has insignificant increment in the mass transfer [21]. Electroconvection is mainly caused by electroosmosis of the second kind. Electro-osmosis of the second kind occurs in overlimiting condition, due to the action of the electrical force upon the non-equilibrium space charge region. Indeed, when a high electric field is applied perpendicularly on the SCR near the membrane interface, an excess pressure arises within the SCR (Figure 10a, step 1). This excess pressure (secondary effect from the electric field) displaces the liquid out from the SCR in tangential direction towards zones with lower pressure, corresponding to zones having lower space charge (Figure 10a, step 2). When moving along the membrane interface, this displaced fluid collides with inertial non-slipped liquid layers (Figure 10a, step 3). The resulting difference in inertia redirects the movement of the fluid towards the depletion solution or solution bulk [21]. This results in the occurrence of two electroconvective vortices rotating in opposite senses and having a major role in the increment of overlimiting current (Figure 10a, step 4). However, it is not yet known if this excess pressure is directly related to the action of the electric force or indirectly by the forced migration of numerous counter-ions under the high electric field, carrying water in their hydration layer (Figure 10b, step 1), and if this excess pressure within the SCR, applied on counter-ion hydration shells, displaces part of their hydration water out from the SCR (Figure 10b, step 2), collides with inertial non-slipped liquid layers (Figure 10b, step 3) to create the vortices (Figure 10b, step 4). Currently, the SCR is considered in its entirety and the electric field is assumed to be applied to this entire region and not to each ion separately. So, this region behaves like a volume on which the electric force is applied. Consequently, the relatively high space charge values of the extended SCR, developed on the depletion side of ion-exchange membranes during their polarization under high electric field, and the extended SCR thickness are the conditions necessary for the occurrence of electroconvection [51,59,70,71].

**Figure 11:**
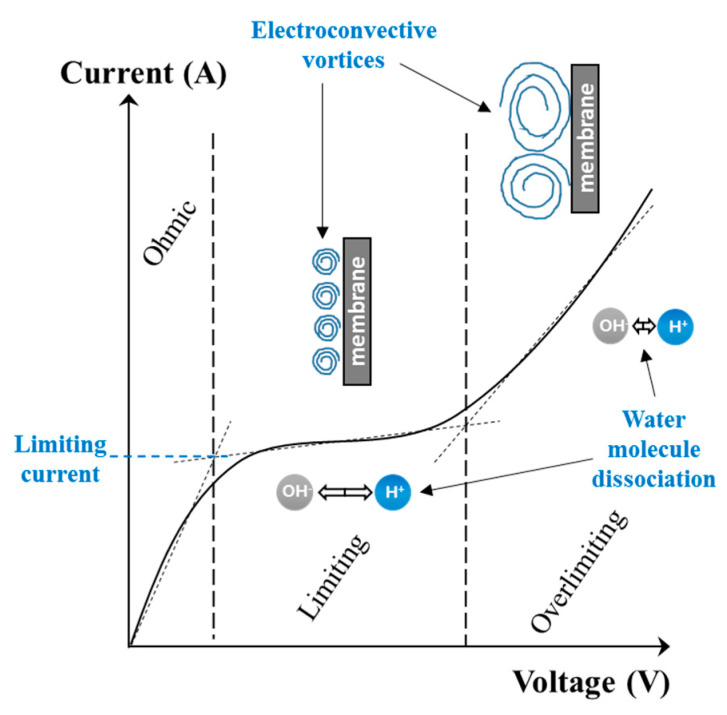
Typical current-voltage curve, appearance of electroconvective vortices and intensity of water molecule dissociation (Adapted from Bazinet and Castaigne [11]).

In addition to mixing the depleted solution at the interface of the membranes with the more concentrated solution in the center of the compartment (bulk solution), these vortices reduce, even eradicate, the diffusion layer. Thus, they reduce mass transfer by diffusion (in the diffusion layer (Figure 4)) which is rather slow and, accelerate the mass transfer by migration (Figure 4). Furthermore, the vortices reduce the phenomenon of dissociation of water molecules by inducing agitation of the solution at the interface of the membrane and thus decreasing the diffusion layer thickness (Figure 11); the total increment of the water splitting in the overlimiting current is really small (around 5%) and this mechanism cannot be considered as a main mechanism [9]. Two types of electroconvective vortexes can be distinguished: relatively small and stable vortexes (low voltage or current intensity, limiting region) and unstable vortexes of larger sizes, appearing at thresholds of 1 V/pair of cells, in the over-limiting region [72] (Figure 11). Contemporary methods of investigations of the electro-osmotic instability such as laser interferometry, flicker noise spectroscopy, particle tracking velocimetry and microfluidic ED platforms coupled with electrochemical methods and modeling allow deeper understanding of this phenomenon [59,73,74,75,76,77]. For more fundamental information and equations about electroconvection please referred to the reviews of Nikonenko et al. [9,51].

The main factors responsible for the development of electroconvection are the electric and/or geometric heterogeneities of the membrane surface [78,79,80,81], the hydrophobicity of the surface and its charge [82], the change in resin/inert binder ratio [77] as well as the concentration distribution heterogeneity at the membrane/solution interface [72]. All these effects are synergistic and contribute to the appearance of the tangential component of the electric force, which sets the volume of the solution at the membrane surface in motion [9,52]. Hence, recently, it was observed that for relatively uniform distribution of widely spaced conducting areas, the vortex size near the membrane surface increased from about 50 to 200 μm for modified membranes; the vortex size being determined by the distance between two neighboring conducting areas [81].

#### 3.4.3. Effects of Overliming Current Conditions on Electrodialytic Process Performances

As demonstrated by recent studies, electroconvective vortices improve ED performances and/or cost by (1) facilitating the transport of ions towards the surface of the ion exchange membrane, (2) decreasing the surface of membrane needed for the same operation and (3) affecting fouling formation. Indeed, the electroconvective vortices have the effect of thinning the boundary layer at the surface of the membrane and thus increasing the mass transfer of ion by agitation: the voltage increase in the ED cell then results in an increase in intensity (Figure 11, overlimiting region) [9]. Besides the improvement in ion transfer, the ED operation with intensive current may reduce the membrane area, which is considerably advantageous, since the costs of ion-exchange membranes are generally high; at an industrial scale, the cost of the IEMs and spacers contributes to 25–30% of the total cost of a new ED unit fully automated, but this relative percentage increases when the level of automation is decreased. On another hand, operating at overlimiting current densities may lead to intense water dissociation at the membrane surface, which favors the deposition of organic and inorganic substances on it, fouling and scaling, respectively [65,83,84]: the cost of cleaning procedures and membrane replacement may vary to 40–50% for the electrodialytic processes [85,86]. However, Bukhovets et al. [87] reported the influence of electroconvection on the prevention of organic fouling by the “washing” effect of the vortices, while Mikhaylin et al. [88] demonstrated the positive effect of electroconvection on the attenuation of scaling on ion exchange membranes.

The overlimiting regime seems to be an advantageous solution to improve the efficiency of ED processes. However, several aspects must still be studied or taken into account before overlimiting current conditions can be used at an industrial scale such as (1) electricity costs and production, (2) energy consumption, (3) heat production by Joule effect and (4) impacts of water dissociation and vortices on the membrane integrity in the long term. Indeed, as demonstrated by Mikhaylin et al. [88], the energy cost for an electrodialytic process depends on the way the electricity is produced (nuclear plant, hydroelectric plant, coal plant, wind, etc.) and the source used (renewable and non-renewable). Concerning the energy consumption of the process in overlimiting condition and the production of heat, the studies are generally carried-out with controlling the temperature of the process, since the temperature of the solution increased rapidly due to the Joule effect and/or the continuous function of pumps. If the temperature of the solution has to be controlled, by the use of a heat-exchanger for example, this will lead to an additional energy consumption. However, it was demonstrated, that for a 62% demineralization of whey, by ED in ohmic conditions, the time required decreased by a factor of 2, the total mass flux increased 3 times whereas specific energy consumption was the same when the temperature was changed from 15 to 55 °C [89]. Consequently, such an induced increase of temperature in overlimiting condition could be a potential advantage. Indeed, as demonstrated very recently by Beaulieu et al. [90], it would improve the transfer of ions. However, according to these authors, the use of an overliming current condition coupled with the temperature increase induced did not improve the energy efficiency of the process. On the contrary, the application of overlimiting condition increased by 7–9 times the energy consumption compared to ohmic condition (for more information see Section 4.1). Santana Barros et al., recently evaluated the impact of underlimiting conditions and concluded that electrodialysis in overlimiting condition seems to be more advantageous than in underlimiting one [91]. However, the authors did not evaluate the energy consumption of both processes (for more information see Section 4.1). Many adverse effects of water splitting at the membrane interface on structural changes of IEM surface used in electrodialytic processes at limiting current density (LCD) condition has also been observed [46,92,93,94]. Enhanced dissociation of water molecules and changes in membrane physicochemical properties in used AEM was noticed, suggesting a gradual degradation of AEM during ED process which consequently increased the surface roughness. Indeed, many anion-exchange membranes intrinsically contain quaternary ammonium groups (–N(CH_3_)_3_^+^), in which the nitrogen atom does not carry any lone electron pair and consequently water splitting cannot occur (see Section 3.3.1, rate constant k_lim_ = 0 s^−1^). Nonetheless in overlimiting conditions, OH^−^ electrogenerated by water dissociation can act upon these fixed quaternary ammonium groups to convert them into tertiary (–NH(CH_3_)_2_^+^) or secondary amine (–NH_2_(CH_3_)^+^) as described by Hofmann degradation (E2), elimination (E1) and nucleophilic substitution [93,95,96,97]. In addition, Choi and Moon [94] noticed this degradation after only one hour under a strong electric field. The generated tertiary amines can further induce the catalytic water splitting by reacting with pre-polarized water molecules by reversible protonation and deprotonation under strong electric field [92,94]. Such degradations of aliphatic quaternary ammonium group to tertiary amines were suggested at LCD and alkaline conditions [46,92,93,96]. In addition, according to Pismenskaya et al. [33] after 10 h of application in overlimiting current conditions and exposure to vortices, cavities would be formed on the surface of the ion-exchange membranes. However, these cavities would occupy only 0.4% of the membrane surface after 100 h of overlimitng current application and would even increase mass transfer. Thus, the effect of overlimiting current on the lifetime of membranes during extended treatment duration comparable at the ones in the industry (between 3000 and 20,000 h) is still unknown.

### 3.5. Pulsed Electric Field Application and Influences on Membrane Phenomena

#### 3.5.1. Principle of Pulsed Electric Field

**Figure 12:**
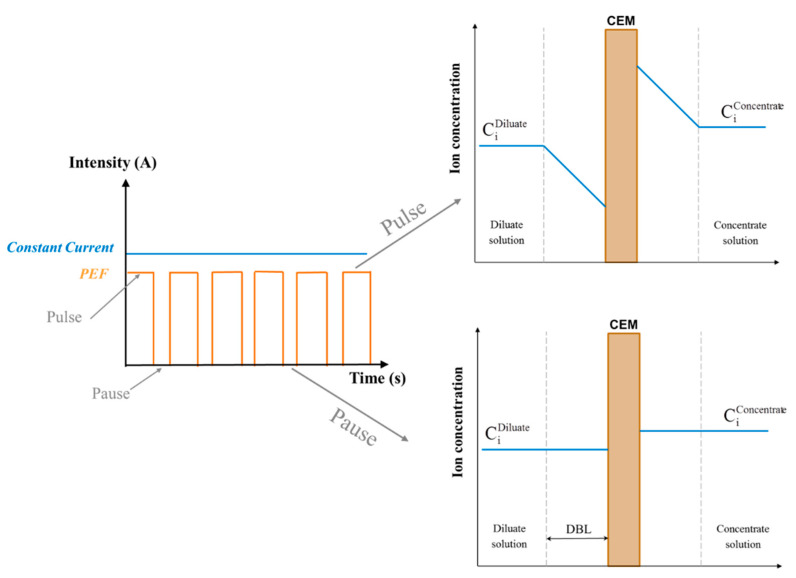
Principle of pulsed electric field (PEF) and effect on concentration polarisation. CEM: cation-exchange membrane; DBL: diffusion boundary layer.

The pulsed mode is a non-stationary regime applying a hashed current (Figure 12) or voltage: for a defined time, the system is under the influence of a constant current/voltage (Pulse lapse—T_on_), then the current/voltage is turned off for a fixed time (Pause lapse—T_off_). Pulsed electric field (PEF) procedure consists in application of consecutive pulse and pause lapses (T_on_/T_off_) of constant duration. The application of PEF in electrodialysis was first proposed by Karlin and Kropotov in 1995 [98].

#### 3.5.2. Advantages and Limitations

PEF has numerous advantages such as (1) suppression of concentration polarization phenomenon, (2) decrease in water dissociation, (3) enhances demineralization process rate and reduce system resistance, (4) increases the current efficiency and consequently decrease the relative energy consumption, (5) enhances transfer of charged biomolecules (organic acid, amino acids, etc.), (6) effectiveness against fouling, (7) induces a selective ion migration according to the pulse/pause combination used and (8) simplicity of equipment, which makes integration of such approach into industrial scale easy and inexpensive but it also currently presents some limitations.

First, the positive influence of PEF was found in terms of decrease in concentration polarization phenomenon and consequently decrease in water dissociation [51,54,99,100]. During each pause time, the concentration polarization decreases until it eventually disappears (Figure 12). This time, necessary for the disappearance of the boundary layer, is called transition time and makes it possible to return to an initial state of concentration similar to that of the homogeneous solution (Figure 12). It mainly depends on the diffusion of ions through the membranes and in the boundary layer, the ion concentration as well as the voltage applied to the system [54,99]. Hence, removing the concentration polarization phenomenon increases the efficiency of the current and therefore of the energy consumed during the process [99]. Recently, the scientific community has gained a better understanding of the phenomena involved during the pauses in PEF electrodialysis. After pausing the system, a low electrical resistance is achieved because of the restitution of ions in the depleted layer at the diluate membrane interface. The re-application of a voltage after each pulse promotes electroconvective vortices (ECVs) that translates into current surges [101]. The electroconvection affects turbulence, even at low Reynolds numbers [78]. ECVs promote the transfer of ions onto the surface of the membranes [51], making it possible to work in overlimiting current conditions under negligible water dissociation [64,65]. It was also proven that mass transfer could be intensified several times in comparison with continuous current (CC), depending on the pulse-pause characteristics [54]. Regarding fouling, recent studies reported the prevention of fouling by application of PEF. Indeed, due to the disappearance of the boundary layer and the regular re-homogenization of the solution around the membranes, the PEFs have proven to be an effective solution to mitigate fouling caused by humate [39,102], proteins [103,104], peptides/amino acids [105], lignin [106], natural organic matter [107], partially hydrolyzed polyacrylamid [84] and mineral scaling [64,65,83,84,85,104,108,109,110,111,112,113] during electrodialytic processes (Table 1). Deep investigations have been carried out to reveal the mechanisms of scaling prevention under the action of PEF [104,109,110,114] as well as fouling mitigation by natural organic matter [107,115,116,117] and lignin [106]. Regarding specific migration of mineral ions, the difference in monovalent and divalent ion demineralization caused by PEF could be explained by several points reported in the literature. First of all, it has been shown that low current density favor divalent ion migration due to their stronger interaction with the membrane’s functional groups [118,119]. Inversely, a higher current density increases the concentration polarization and favors the separation of monovalent ions due to their higher diffusivity in the membrane’s boundary layer. During pause duration, the diffusion layer is rapidly enriched in ions and the concentration polarization is reduced, but a potential difference is preserved and a slight electro-osmotic movement towards and through the membranes persists in the solution [54]. Hence, divalent ions such as calcium and magnesium will preferentially interact with the membrane’s ion-exchange groups due to their higher sorption coefficient [120] (for more explanations see Section 4.2.1). Finally, the equipment needed for the generation of pulse-pause from a generator is quite simple, via the use of a pulse generator coupled to the power supply or the automatic pulse generation via a computer controlling the power supply. Consequently, the simplicity of such a piece of equipment and their low cost should favor the introduction of PEF at large scale.

Despite its simplicity and flexibility, and since no special anode is required in comparison with EDR, PEF is not as intensive for self-cleaning. Moreover, the total working time of the ED system is increased by pausing the system. The only way to have a similar working time as for CC to run a specific process is to increase the membrane area and number of cells needed. Such a drawback has not been considered yet in the literature, which is a critical factor in desalination or other ED applications such as EDBM. Indeed, the operation time directly affects the economy of the processes at industrial scale. However, very recently, Lemay et al. [65] demonstrated for the first time that a high frequency PEF condition of 5 Hz required almost the same time, including the PEF pause duration, than CC condition to reach the same final demineralization rate and that with a lower energy consumption (for more precisions see Section 4.2.1). In addition to the points highlighted before, to fully evaluate the potential of PEF application, it will be important to study the membrane integrity after long use periods of PEF, following electroconvective appearance and water dissociation phenomena as mentioned previously in Section 3.4.3 for overlimiting current conditions. Finally, it is worth to mention that the application of PEF, at a larger scale, will depend on the availability of power sources designed to be continuously switched on and off.

#### 3.5.3. Is There One Optimal PEF Condition?

The choice of PEF parameters (pulse/pause durations, ratio and frequency) is, however, delicate and significant differences between processes are visible depending on the parameters chosen (Table 1). Indeed, the effects of variations in the duration of each of the two periods, their ratio as well as the frequency of PEF has been highlighted during electrodialytic treatment for fouling mitigation, increasing energy efficiency and improving mass transfer. The following analyses are based on data available in the literature and whose authors have tested at least three different PEF conditions to find potential trends.

##### For Fouling Mitigation

**Figure 13:**
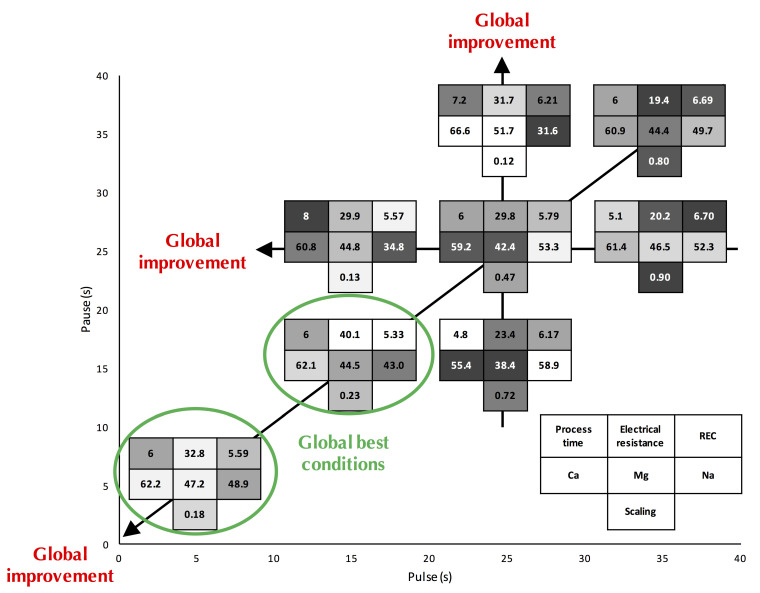
Schematic representation of the studied surface area comprised in the eight PEF conditions tested. Each condition is represented by a table including in the first row: its process duration (in h), its final electrical resistance reduction from the CC application results reported by Dufton et al. [113] (in %) and its relative energy consumption (in Wh/g of lactic acid removed). The second row includes the demineralization rates of calcium, magnesium and sodium (in %), while the third row report the amount of scaling on the AEMs (in g/100 g of dry membrane). The shades of grey represent the position of the condition among the others: the lighter the grey, the better the result in term of acid whey treatment (adapted from Dufton et al. [83]).

In the case of protein solutions [103,104], peptides [105], lignin [106], and organic fouling [84], it was found that the use of long pauses during the application of PEF could remove macromolecule fouling at the surface of IEM (Table 1). This positive effect of a longer pause duration is explained by the better detachment of macromolecules from the membrane surface and/or electrostatic interaction decrease by the solution flux due to the washing effect. Regarding solutions with divalent potential scaling ions [110], the application of pulse/pause regimes with ratio 1, 10 s/10 s was proved beneficial for decreasing scaling formation. However, no entire scaling prevention was obtained due to the strong interactions of scaling ions with membrane ion-exchange groups. Subsequent studies conducted by Mikhaylin et al. revealed that short pulse duration in PEF mode (between 1 and 3 s) allow better mitigation of concentration polarization and scaling phenomena [112]. Indeed, the application of the optimal pulse/pause mode of 2 s/0.5 s allows a 40% decrease of the CEM scaling and complete inhibition of AEM scaling (just traces of AEM scaling were detected) in comparison with a continuous current mode [112]. The working principle behind the effectiveness of PEF, for scaling mitigation, is related to reducing concentration polarization, in this case, to avoid the accumulation and close packing of foulants at the surface of the membrane combined with the cleaning effect of the solution recirculation. Very recently, on more complex solutions, such as polymer-flooding produced water and acid whey, the impact of the pulse/pause durations was also demonstrated (Table 1) and the best conditions were between 0.01 and 0.25 Hz. Hence, for polymer-flooding produced water [84], the authors pointed out that the 10 s/30 s and 1 s/3 s regimes, same ratio of 3, were the best for mitigating the negative effects of concentration polarization and organic fouling. For acid whey, it was demonstrated that decreasing the pulse/pause durations from 35 s/35 s to 5 s/5 s, same ratio of 1, decreased drastically the scaling (77% less scaling) (Figure 13). Furthermore, decreasing the pulse duration from 35 s to 15 s for a same pause duration (35 s) led to a scaling decrease of 85% while decreasing the pause duration from 35 s to 15 s for a same pulse duration of 25 s, increased the scaling by 6 folds [83] (Figure 13).

It appears that there is no real consensus according to PEF parameters to apply in order to eliminateor decrease the fouling or scaling of IEM, as well as the type of parameter to be taken into account (pulse and pause durations, pulse/pause ratio or frequency). However, from all the studies reported in the literature, short pulse and pause duration as well as ratio around 1 would be more effective on scaling while short pulses, with long pause would be more effective for eliminating the different types of fouling (protein, lignin, peptide, etc…). Concerning optimum frequency, the use of pulsations with a frequency of the order of 0.1 Hz and an extended relaxation time would allow the mitigation of fouling. However, the optimum frequency for fouling mitigation is depending on the membrane structural difference [102].

##### For Energy Efficiency

**Figure 14:**
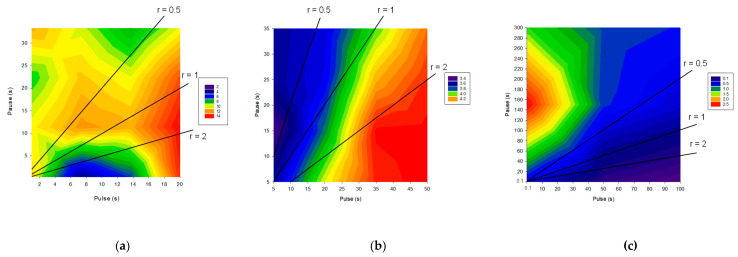
Two-dimensional contour plots of relative energy consumption (Wh/1000 C) as a function of pulse and pause durations for (**a**) demineralization of model salt solution (adapted from [66,110,111,112]; (**b**) demineralization/lactic acid recovery of acid whey (adapted from [83,113] and (**c**) demineralization of polymer-flooded produced water (adapted from Sosa-Fernandez et al. [84]. r = pulse duration/pause duration ratio.

Beyond the reduction in fouling, the pulsed electric field parameters have also major influence on the global process energy efficiency. However, very few studies compared systematically the impact of the pulse and pause durations, pulse and pause ratio and/or frequency as well as calculation of the energy consumption of the same basis to be able to find the optimum conditions in terms of energy consumption (Table 1). Hence, on Figure 14, the relative energy consumption (REC) from different studies and for different solutions was expressed in Wh/1000 C in order to compare these processes. Here, the representation of the REC as a function of pulse and pause durations was chosen, since REC as a function of frequency or ratio did not give any conclusive results. It appeared that the optimum REC zone is different according to the solution to be treated. Indeed, for model salt solutions containing a magnesium/calcium ratio of 2/5, close to the mineral composition of milk, the best conditions appeared to be pulse durations between 6–10 s with pause durations lower than 1 s (corresponding to pulse/pause ratios over 6 and frequencies lower than 0.16 Hz). For these solutions the optimum combination seems to be in a quite narrow zone. For more complex solutions containing not only salts, the optimum zone seems to be wider. Hence, for acid whey the best conditions would be with pulse durations lower than 10 s, or more specifically pulse durations lower than 10 s and pause durations around 15 s (corresponding to pulse/pause ratios lower than 1 and frequencies higher than 0.04 Hz) and for polymer flooding produced water with pulse durations over 50 s, or more precisely pulse durations over 50 s and pause durations lower than 20 s but over 0.1 s (corresponding to pulse/pause ratios higher than 2.5 and frequencies lower than 0.02 Hz). On acid whey demineralization and lactic acid recovery, the energy gain would be due to the cumulative effect of scaling mitigation and lower ohmic resistance of the diffusion layer after the pause lapse. This allowed, for a short moment after current re-establishment, a concentration polarization higher than the one normally allowed by the actual limiting current density. This gain was more visible at higher frequencies such as the ones used by Sistat et al. [121] on model salt solution and Lemay et al. [64] on sweet whey.

For the energy consumption, no general trend can be drawn, and opposite trends are even observed. The optimum PEF conditions, in terms of energy consumption, seem to be dictated by the composition of the solution as well as the potential fouling that PEF can mitigated. The impact of PEF on fouling mitigation cannot be dissociated from the global process performance where energy consumption is one of the main components.

##### For Mass Transfer

**Figure 15:**
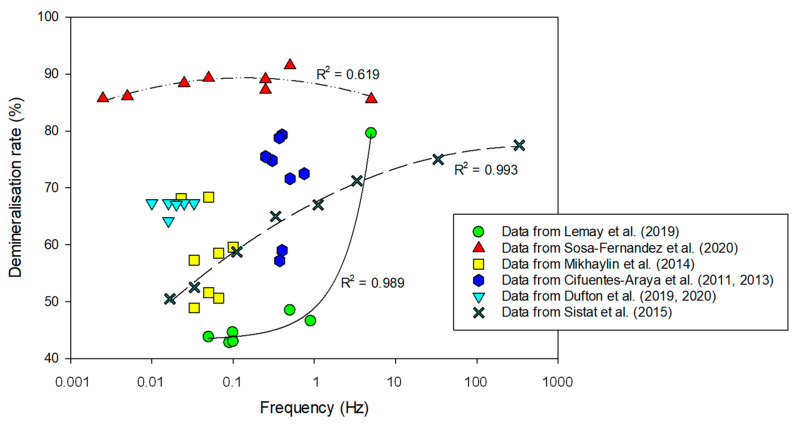
Impact of frequency (logarithmic scale) on the demineralization rate (in %). Data were obtained from studies reported in the literature and working with different conditions and solutions: model salt solutions [110,111,112,121], acid whey [83,113], sweet whey [64], polymer flooding produced water [84].

PEF was also tested in the context of mass transfer for improving demineralization or/and organic acid recovery as well as divalent cation selectivity. Concerning demineralization, it was reported on polymer-flooding produced water that PEF improved the demineralization percentage and according to the authors, the shorter the pulse duration, the higher the demineralization rate [84] (Table 1). However, the only exception was for the runs with 0.1 s pulses, which rendered low demineralization percentages, possibly due to the high frequency that could cause a closest packing of the foulant: partially hydrolyzed polyacrylamide (HPAM). For regimes with the same pulse and different pause period, longer pauses yielded lower demineralization of the stream. For a feed stream consisting of brackish water + HPAM, the best demineralization rate was obtained employing the regime 1 s/1 s [84]. This is confirmed on Figure 15 representing the demineralization rate as a function of frequency (on a logarithmic scale) and where the condition 1 s/1 s corresponds to a frequency of 0.5 Hz, and is the optimum. On the opposite, on sweet whey demineralization, the highest demineralization rate was obtained for PEF combinations 0.1 s/0.1 s with a demineralization rate 81% superior to CC mode for the same number of charges transported (Figure 15). It is important to mention that PEF application of 1 s/1 s condition was the second-best condition, however with a demineralization rate only 10.5% higher than CC [64] (Table 1). This was explained by the emergence of electroconvective vortices (ECVs) at the beginning of each pulse due to the appearance of a voltage spike. Since their lifetime are around 0.5 s, these ECV did not have time to fade off during the whole process in the case of high-frequency PEFs increasing consequently the mass transfer [65] (for more explanation see Section 4.2.1). On acid whey demineralization and lactic acid recovery by ED, it was observed that the combination of pulse durations between 5–50 s and, pause durations between 5–35 s led to a similar global demineralization rate around 67% [83,113] (Table 1). Following the results of simulation compared with experimental data obtained on a laboratory-scale electrodialysis stack, with a 0.1 M NaCl solution, Sistat et al. showed that mass transfer under PEF of a sufficiently high frequency (>1 Hz) was higher than the one in conventional steady state direct current (DC) mode, if a same average voltage is applied [121]. The advantage increases with frequency and reaches a plateau at about 100 Hz (Figure 15). The authors explained these results by the fact that when applying a pulse after a pause, there are low ohmic resistance and low diffusion potential drop caused by partial concentration restoration in close vicinity of the membrane beneficial to the mass transfer. This allows passage of an instantaneous current of a high density, which can essentially exceed the limiting current density in steady state DC conditions. However, at low frequencies this gain is rapidly vanished by increasing concentration polarization during the pulse; thereby the mass transfer in PEF mode is lower than that in DC mode. Although these experiments were carried-out with a model solution containing only 0.1 M NaCl and a duration of each experimental run of 10 min, a similar conclusion was obtained by Lemay et al. [64] on sweet whey (Figure 15). Indeed, the authors reported on this complex food solution that the mass transfer efficiency increased with the frequency of the pulse/pause combination; 5 Hz (0.1 s/0.1 s) being the best condition (Figure 15) (for more information see Section 4.2.1). However, recently, Dufton et al. [83] on demineralization and recovery of lactic acid from acid whey observed that an overall enhancement of the ED process was visible through the increasing of frequency, even for frequencies comprised between 0.014–0.1 Hz. So, the beneficial impacts of frequency were not only visible for high-frequency PEFs (higher than 1 Hz) in regard to mass transfer improvements.

Recently, during the recovery of citric and malic acid from cranberry juice by EDBM, it was demonstrated that pulse durations of 1 and 2 s with pause durations higher or equal to 1 s were optimum [122] (for more information see Section 4.2.2). The authors also observed that the pause duration should be increased when the pulse duration is increased. According to these authors, decreasing the DBL thickness entirely, or to a sufficiently low value, allowed better migration of anions of organic acid, but a pause duration equal or longer than 1 s is needed. Furthermore, when the pulse duration is increased, the pause lapse must also be increased. On the opposite, similar lactic acid recovery rates were observed during the acid whey demineralization and lactic acid recovery by ED, when combination of pulse durations between 5 s and 50 s and, pause durations between 5 s and 35 s were tested: a lactic acid recovery rate of 44.5% was reported [83,113] (Table 1). Such a difference would be due to the different migration kinetics of the ionic species present in the solution, since in the first case, mainly the anionic forms of citric and malic acids from cranberry juice are migrating through the AEM while there was a competitive migration between chlorine, H_x_Cit_y_^n−^ and H_x_P_y_O_z_^n−^ amongst other salts present in acid whey and the anionic form of lactic acid. Due to the high concentrations of salts and the difference in electrophoretic mobility between salts and anionic forms of organic acids, the effect of PEF is less effective or observable for acid whey.

Concerning divalent ion selectivity, it was reported that the longer the pause duration, the stronger the competition between calcium/magnesium and sodium/potassium at the membrane’s ion-exchange groups and the higher the migration of divalent ions [65,83]. The higher migration rates of Ca^2+^ and Mg^2+^ observed for PEF condition with longer pause lapse was explained by the partial restoration of (1) the concentration profiles in the solutions adjacent to the membrane surface and (2) the membrane control over the kinetics of ion separation [65] (see Section 4.2.1 for more explanations).

For mass transfer, it seems that a short pulse duration (around 1 s) associated with a short pause duration (around 1 s) and corresponding to a ratio of 1 or a frequency over 1 Hz but lower than 100 Hz would be the best conditions.

##### Global Process Efficiency

It appears from all the previous information that the optimal conditions are different from one solution to another and from one component of the global process efficiency (fouling/scaling mitigation, energy consumption or mass transfer) to another. Hence, to optimize the PEF condition, more than one component should be taken into account, since these components are linked. Indeed, if a fouling appears rapidly at the beginning or during the process, as with the use of BPM generating H^+^ (inducing protein precipitation) and OH^−^ (inducing lignin precipitation or scaling), the energy consumption as well as the demineralization rate or more generally the mass transfer will decrease due to the current efficiency decrease. In this case, the fouling/scaling mitigation will first dictate the choice of PEF conditions, to allow the process being carried out [106]. If no major fouling appears, drastically slowing down the process, the choice will be dictated by the energy consumption related to the species of interest for reaching the desirable migration rate. However, in this second case, a fouling or scaling can appear during the process, due to the LCD reaching, linked with (1) a high demineralization rate for example (generation of H^+^ and OH^−^ at the IEM inducing fouling/scaling) or/and (2) the solubility constant of a species related to its high concentration at the IEMs interface due to the membrane permselectivity (inducing its precipitation and scaling). Indeed, for scaling/fouling that could appear during the process, and decrease the global efficiency of the process for consecutive runs or if no sufficient or not adapted cleaning-in-place procedures are used, the choice of PEF conditions can be completed taking into account its impact on ECVs and the polarisation concentration phenomenon. In fact, the best conditions would be a pulse duration over the duration of vortex appearance (about 0.1 s [51]) and lower than the concentration polarization formation (about 1 s [66,101]) and a pause duration lower than the duration for vortex disappearance (about 0.5 s [112]) as demonstrated by Lemay et al. [64,65]. Hence, in sub-limiting conditions, such a condition of PEF will favor the presence of vortices, eliminating the concentration polarisation phenomenon as well as diminishing the water splitting appearance when high demineralization rates are aimed and consequently the possibility of scaling or fouling. In parallel these conditions would maintain or increase the relative energy consumption by increasing the mass transfer enhanced by the constant presence of vortices. Such conditions would then take advantage of the overlimiting conditions (vortices, decrease in polarisation concentration and increase in mass transfer) without the inconvenient linked to a greatly higher relative energy consumption [90].

## 4. Recent Technological Developments Based on ED Membrane Phenomena

While ED is steadily increasing its influence over the market of pure water production, researchers and engineers are still pursuing ways for improving its performance. To that end, new technologies derivate from conventional ED are developed and lead to unveiling new types of applications usually complementary to pure water production. This part is dedicated to the latest advances (applications of electroconvection phenomenon, pulsed-electric field, technological improvement or design…) reported for ED technologies presented in Figure 1. Aiming at presenting ED membrane phenomena-based processes, some technologies were not detailed: membrane-free electrodeionization [123], electrostatic shielding electrodeionization [124] used for metal ion removal, microbial desalination cell for wastewater treatment, pure water and energy production [125] and redox flow battery for energy production [126]. Furthermore, the lack of recent developments led us to not detailed electro-ion injection-extraction (EIIE), electro-ion substitution (EIS) and electrometathesis (EMT) [127,128,129], mostly used for the recovery of organic acids.

### 4.1. ED in Overlimiting/Electroconvective Conditions

Very recently, the application of ED in electroconvective conditions was evaluated on complex solutions. Hence, Santana Barros et al., evaluated the impact of underlimiting and overlimiting current conditions during ED of a cyanide-free wastewater from brass electrodeposition [91]. According to these authors, the water dissociation phenomenon, the reaction of protons with complexes and insoluble species, the intense electric field and the electroconvection may have allowed the migration of the co-ions Cu^2+^ and Zn^2+^ through the CEM, favoring their extraction. The overlimiting phenomena accounted for the improvement of the percent extraction and percent concentration, since the concentrate compartments of the electrodialysis stack were connected to the same reservoir. Chronopotentiometric studies showed also that electroconvective vortices minimized fouling/scaling at both membranes. According to the authors, small intermembrane distances are recommended, since the intermembrane distance plays an important role in ionic transfer when water dissociation is dominant. They concluded also that electrodialysis in an overlimiting condition seems to be more advantageous than in an underlimiting one, but they did not evaluate the energy consumption of both processes [91]. In addition, on acid whey demineralization and lactate recovery, Beaulieu et al. [90] studied the impact of electroconvective vortices on ED process efficiency. They compared the ED process both in underlimiting and overlimiting current conditions in terms of demineralization, lactate recovery, ion migration, temperature increase, overall system resistance and energy consumption. The results showed that overlimiting current conditions led to a higher demineralization (87% based on the total cation concentration) in half the time (30 min at 3 A vs. 60 min at 0.7 A) while the migration of lactate (around 33%) was not enhanced or improved by the overlimiting condition and the formation of ECVs. ECVs are known to shrink the laminar boundary layers as well as to reduce or eliminate the concentration polarization at the membrane interface, leading to the release of current carriers and a better mass transfer of ionic species [21,54,66], however, all the main studies reported in the literature were carried out on model solutions, and solutions containing organic acids had never been tested. Thus, it appeared from these results that the mass transfer of organic acid seemed to be not improved by electroconvective vortices in contrary of mineral species. Indeed, the production of H^+^, in the whey compartment at the interface of the AEM after the LCD was reached would have impacted the migration of the lactate due to its pKa of 3.86 to produce non-charged lactic acid, decreasing its potential to migrate. Concerning ion migration, whatever the condition of current applied, the potassium was the ion having the highest migration rate related to its high electrophoretic mobility and concentration. Furthermore, as already observed in the literature, a higher current density favors the concentration polarization and increases the migration of monovalent ions, which is related to their higher diffusivity in the membrane’s boundary layer [83,118,119]. This explains why calcium and magnesium closely followed potassium with similar migration rates in the overlimiting condition. However, in the underlimiting condition, magnesium had a smaller migration rate (41.4% vs. 51.2% and more for other cations) that can be explained by its slower mass transfer [65,130] and the absence of ECVs that can enhance its migration and all other cations [21,64,65,66]. Finally, concerning the energy consumption, it was reported that the application of overlimiting condition increased the REC, compared to ohmic condition, during lactic acid recovery and ion removal by electrodialysis: the increase in current was 4.3-fold, but the energy consumption increased respectively by 8–11 folds.

### 4.2. Application of PEF during ED

#### 4.2.1. ED-PEF

The application of PEF on complex solutions (Ion-exchange brine solution, wastewater, food by-products, beverages, etc.) more close to real industrial applications is recent and mainly benefits from the advantages of energy consumption and improved transfers.

When natural organic matter (NOM) is removed by ion-exchange (IX) via anionic IX resin from potable water for aesthetic, operational and indirect health concerns, it generates spent brine. To overpass this main drawback related to the strict discharge regulations and limited and costly brine management options, ED-PEF desalination of the IX brine was tested recently [107]. The highest demineralization rate (95.5%) was achieved when the IX spent brine was treated under the PEF regime with conventional permselective AEMs instead of monovalent permselective AEMs (AMX vs. ACS) and the PEF regime was efficient in intensifying the desalination process only when the conventional AEMs were used. However, desalinating the IX spent brine with AMX membranes and under PEF regime resulted in a higher sulfate content in the produced NaCl solution. By drawing a correlation between the AEMs characterization data and evolution of the electrodialytic parameters, the authors deducted that fouling and/or adsorption of low molecular weight acids and low molecular weight neutrals fractions of NOM deteriorated AMX membranes. However, during the three consecutive runs the characteristics of the IEMs and applied electric field (CC vs. PEF) had no significant impact on the relative energy consumption values (≤2.1 Wh/g NaCl). Polymer-flooding produced water is also an abundant by-product from the oil and gas industry, with potential reuse after partial demineralization [131,132]. To avoid concentration polarization and fouling which hampers the desalination of polymer-flooding produced water (PFPW) by electrodialysis, Sosa-Fernandez et al. carried out an extended study on the application of eight different PEF conditions [84] (Table 1). They demonstrated that the application of PEF improved the ED performance in terms of demineralization (around 9% for PEF 1 s/1 s in comparison with CC mode of current) and energy consumption (36% reduction for PEF 1 s/3 s). When comparing the performance of the 1 s/1 s and the 1 s/3 s regimes, the gain in energy consumption for 1 s/3 s was demonstrated higher than the gain in demineralization for 1 s/1 s. However, the regime 1 s/3 s also implies that the membrane stack is effectively in use only 25% of the time. Considering all these factors, they concluded that the most promising of the regimes would be 1 s/1 s. They also claimed that in general, the shorter the pulses, the higher the demineralization rate and the lower the energy consumption. The only exception was for the runs with 0.1 s pulses, which rendered low energy consumption but also lower demineralization percentages, possibly due to the high frequency that could cause a closest packing of the partially hydrolyzed polyacrylamide [39].

Two by-products produced in very large quantities, by the dairy industry, all around the world are sweet and acid wheys. Lemay et al. [64] reported that a sweet whey demineralization treatment with 2 h of applied current led to an increased mass transfer efficiency with the frequency of the pulse/pause combination; 5 Hz (0.1 s/0.1 s) being the best condition. For acid whey demineralization (3 h treatments), the best PEF conditions providing the highest cumulative removal of Ca^2+^, Mg^2+^ and Na^+^ were 5 s/5 s and 15 s/15 s [83]. In addition, the migration of lactic acid from acid whey was accelerated by 16% and the energy consumption decreased by 33% for PEF condition of 25 s/25 s [113]. The higher migration rates of Ca^2+^ and Mg^2+^ observed, during whey treatment by electrodialysis for PEF conditions with longer pause lapse, was explained by the partial restoration of (1) the concentration profiles in the solutions adjacent to the membrane surface and (2) the membrane control over the kinetics of ion separation [65]. Indeed, high electrostatic interaction of the divalent ions with the fixed ions essentially reduces their mobility in the membrane. However, at low current densities, the factor of higher affinity of calcium (and magnesium) is the dominant one, and sulfo-cation-exchange membranes are preferably permeable for the doubly charged cations [133,134,135]. Nevertheless, this specific permselectivity towards doubly charged counter ions is lost with increasing current density [133,134,136]. In PEF, a partial restoration of the specific permselectivity is observed. The reason of partial restoration of the specific permselectivity is that, during the pause lapse, there is a partial reconstitution of the concentration profiles in the solution adjacent to the membrane surface [65,99,101,121]. That is, the concentration profiles near the membrane interface tend to restore their shape corresponding to zero current density. With this, the membrane also partially restores the control over the kinetics of ion separation. Furthermore, the remnant vortices during the pause lapse would contribute in mixing the near-surface solution, hence, in a quicker restoration of the concentration profiles. In addition, the ECVs developed near the membrane during the pulse of current should mix the near-surface solution and to maintain a higher solution concentration at the surface. To confirm this hypothesis of selective transport of divalent ions under PEF, a model based on the non-stationary 1D Nernst–Planck and Poisson equations was proposed by Lemay et al. [65]. The computation using the model gives the following values of the effective transport numbers for Ca^2+^ and Na^+^ at *j* = 0.6 *j*_lim_: 0.52 and 0.48 in the continuous current mode, and 0.68 and 0.32 (respectively, for Ca^2+^ and Na^+^) in the PEF mode at frequency equal to 0.5 Hz.

In addition, in 2020, Lemay et al. [65] demonstrated for the first time, during demineralization of sweet whey, that a high frequency PEF condition of 0.1 s/0.1 s (5 Hz) required almost the same time, including the PEF pause duration, than continuous current condition to reach a final demineralization rate of 70% and that with lower number of charges transported, energy consumption and pH variations than for continuous current condition. As mentioned before in Section 3.4.2, the duration of PEF process is a crucial point.

#### 4.2.2. EDBM-PEF

PEF has been tested for the first time on bipolar membrane by Mikhaylin et al. in 2016 for the precipitation of milk casein to decrease protein fouling and scaling [88]. Since 2016, the PEF during EDBM process has been tested to increase the efficiency of the process in terms of scaling/fouling elimination [88,106], process technical feasibility [106], energy efficiency [122], migration improvement [122] and production of ingredients with new properties [137].

Pelletier et al. [122] reported that the recovery of citric and malic acids, from cranberry juice was about 15–18% faster using PEFs and the energy consumption decreased by 7–10% for 1 s/1 s and 2 s/2 s conditions in comparison to continuous current conditions. Furthermore, the concentration of quinic acid and phenolic compounds remained constant in the feed warranting a preserved quality of the juice. EDBM-PEF used at optimal 2 s/0.5 s PEF conditions [112], demonstrated substantial improvement in limiting scaling during casein production. It maintained a satisfactory permselectivity for CEMs, thus preventing the leakage of hydroxide anions from the base compartment [88]. Electroacidification of black liquor from pulp and paper industry to pH 9.7 by EDBM-PEF was found optimal for 6 s/24 s conditions due to the 80% current efficiency and 2.6 kWh/kg NaOH energy consumption obtained [106]. As a comparison, in DC mode, fouling was observed leading to a 45% current efficiency and 4.6 kWh/kg NaOH power consumption.

#### 4.2.3. EDR-PEF or pEDR

Taking into consideration the recent reports on ECVs, and given the challenges still present in PEF electrodialysis, a new technology was recently proposed by Gonzalez-Vogel and Rojas by the application of asymmetric bipolar (reverse polarity) pulses [138]. Indeed, ED intensification was introduced by application of asymmetric pulses of reverse polarity and very short pulses (10 to 100 μs) at high frequencies (from 100 to 4000 Hz) were tested, modulating the amplitude of the pulses to adjust energy consumption. Under such conditions, the LCD increased by up to 1.6 times compared to conventional ED. Additionally, the pH was kept stable during the whole desalination process, decreasing the occurrence of detrimental effects caused by pH changes. Desalination performance under overlimiting conditions exhibited improved mass transfer, while decreasing the demineralization time by 43%. However, the energy consumption under overlimiting current conditions increased by around 56% or 14% compared to conventional ED at sub-limiting or overlimiting current regimes, respectively. The authors also observed that while transport of ions in PEF mode is dominated by concentration gradients, in pEDR the transport would mainly occur by migration. Hence, by favouring migration, the transport of counter and co-ions during pEDR is promoted by the electric field towards the ion depleted layer, accelerating the refilling process and inducing the formation of electroconvective vortices after re-application of forward voltages.

According to the authors, this new desalination strategy is expected to decrease the capital costs of ED plants given the prospects of operation under reduced equipment sizes [138]. The higher energy consumption appeared clearly as a drawback of pEDR, however, they proposed to use cheap energy such as solar power for small plants to allow water demineralization in an intensive way.

### 4.3. Electrodialysis Metathesis (EDM)

The metathesis reaction consists in the exchange of anions (A, A’) and cations (C, C’) between two salts resulting in their conversion in different salts:

CA + C’A’ => CA’ + C’A
(19)

**Figure 16:**
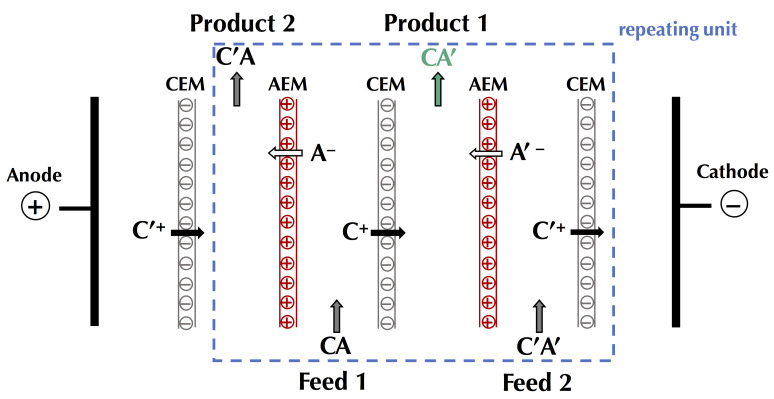
Principle of electrodialysis metathesis. CEM: cation-exchange membrane; AEM: anion-exchange membrane; A^−^, A’^−^: anions; C^+^, C’^+^: cations.

Electrodialysis metathesis (EDM, EDm or ED-M), sometimes also referred to as metathesis electrodialysis (mED) is using two feeds flowing in distinct compartments, each containing a salt to be converted. This technology is taking advantage of the IEMs segregating the compartments to selectively transport a cation from one feed and an anion from the other in order to form a new salt in the adjacent compartment (Figure 16). In addition, EDM generates another new salt due to the transport of ions either from a feed and the electrolyte or from both feeds when several product/feed/product/feed compartment sequences are repeated [139]. While metathesis applied to ED was first discussed in the late 1950s, practical applications at lab/semi-pilot scale go back to the mid-1980s–early 1990s [140]. Early applications of EDM consisted of production of potassium carbonate [141] and iminodiacetic acid involved in the synthesis of the herbicide, glyphosate [142].

Current technological advances make EDM steadily gain more and more interest. Global environmental and resources issues shifted the interest toward EDM compared to less environmental-friendly chemical methods, due to the high purity of its products and its high current efficiency [143,144]. In order to reach optimum conditions in terms of highest current efficiency and product purity, researchers have been continuously studying process parameters. Hence, it was reported that high voltage difference across the stack, sufficiently high feed concentration, equimolar ratio of reactant salts, high membrane transport number and moderate flow rate are critical parameters to obtain ideal operating conditions [144,145,146,147,148,149]. The difference in concentration across the membrane is also of particular importance, since it may result in co-ion fluxes leading to impurities in the final product; monovalent cations are especially subject to this phenomenon [150]. On another note, IEM characteristics strongly influence EDM performance. Indeed, high ion-exchange capacity, low-water content and homogeneous membranes lead to better results in terms of product yield and purity, current efficiency and energy consumption. While they were not optimal for limiting water transport and back diffusion, the overall low impact of those parameters could be further improved by increasing current density and feed concentration [146].

Most recent applications include chloride-free fertilizer synthesis: potassium sulfate [145,151], potassium nitrate [143,146,148,151,152] or potassium carbonate [151]; production of high-value fine chemicals: quaternary ammonium hydroxide reactants and solvents such as, tetrapropyl ammonium hydroxide (TPAOH) [144] or ionic liquid, choline dihydrogen phosphate [147]. While EDM is a relatively low-cost process, additional drying steps (usually, evaporative crystallization) would be required to obtain a solid salt and could impact the final cost [147]. However, it would represent about half the cost of the EDM process and less than 10% of the final product cost in the case of potassium nitrate production [148]. Although known for their high cost, IEMs would only represent around 25% of the total investment in the EDM process [148,151]. Several studies determined suitable economic feasibility at lab scale [144,147,148,151], but EDM has also been successfully implemented at pilot scale as part of a Zero Liquid Discharge Desalination (ZELDA) project as well as in combination with reverse osmosis (RO) for water purification, leading us to expect further developments of this technology in the near future [153,154].

### 4.4. Selectrodialysis (SED)

**Figure 17:**
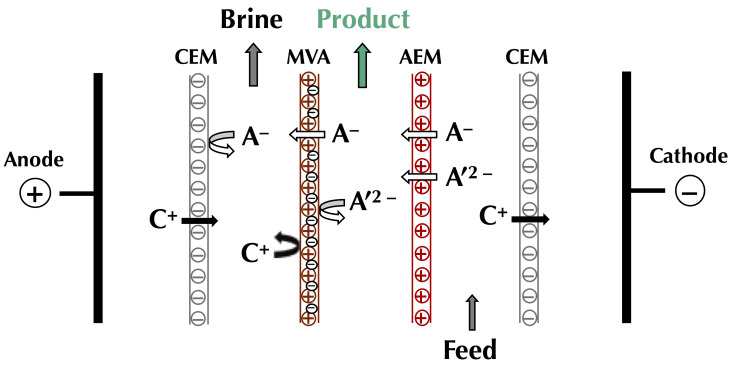
Principle of selectrodialysis. CEM: cation-exchange membrane; AEM: anion-exchange membrane; MVA: monovalent selective anion exchange membrane; A^−^, A’^2−^: anions; C^+^: cations.

Originally developed for wastewater recycling, selectrodialysis (SED), also referred to as selective electrodialysis, is a novel ion fractionation technology leading to better selectivity than conventional ED, ion-exchange (IX) and nanofiltration (NF). Although available at large scale, IEX and ED are more effective for complete and indistinct purification of ions (monovalent and multivalent), while NF allows separation of multivalent ions, but of limited purity in regard to industrial applications [155]. In addition to conventional CEMs and AEMs used in ED, SED process also includes monovalent permselective cation-exchange (MVCs) or anion-exchange membranes (MVAs) to concentrate high-purity multivalent cations or anions [139,155,156]. As illustrated in Figure 17, SED can purify a feed stream containing cation C^+^ as well as monovalent anion A^−^ and divalent anion A’^2−^ by selectively transporting cations through a CEM to a brine compartment and anions through an AEM to an anionic product compartment. There, monovalent anions can be further removed from the anionic product compartment through an MVA to the brine compartment. A similar configuration except for using a MVC adjacent to a cationic product compartment would lead to the purification and recovery of multivalent cations. Along with the purification of multivalent ions, their removals also make SED an adequate process in order to prevent scaling in desalination applications. However, when dealing with natural feed streams (seawater or municipal wastewaters) or industrial feed stream (whey, milk, etc.), the efficiency of MVA and MVC and SED as a whole can be limited due to several factors. Namely, concentration ratio between monovalent and divalent ions, higher proportion of monovalent ion decreases monovalent selective membrane efficiency, and related to this, back diffusion of monovalent ions from highly concentrated brine toward the less concentrated product compartment [157]. Moreover, a small content in multivalent ions tends to increase the global resistance of the SED stack, while current efficiency decreases, resulting in substantial energy consumption [158]. In addition, the concentration in divalent ions should not exceed 40 ppm to preserve the selectivity of the membrane, which is why such a technology is (for example) not adapted for dairy fluids [159]. Furthermore, it was demonstrated that an excessive concentration of multivalent ions (especially cations) increases the risk of scaling on both IEMs and monovalent selective ion-exchange membranes at high pH or when large variation of pH to occur [160]. Finally, when aiming at the recovery of a particular multivalent anion or cation, competition can occur with other co-ions. Depending on the hydrated size of the co-ions, recovery of the targeted multivalent ion can be slowed down, however after sufficient operating time, SED is able to produce the same selectivity as when running a feed solution containing no competing species [161].

Current applications of SED are mostly dedicated to nutrient recovery from wastewaters or effluents, especially, phosphorus [156,161,162], as phosphate anion at 76% recovery but only 44% purity [163] or 93% recovery when combined with a struvite reactor [164]. More recent applications include the recovery of divalent cations such as Mg^2+^ with 75% purity [165] or Zn^2+^ and Cu^2+^ with 80% and 87% recovery, respectively and 99.8% purity for both cations in the product stream [157]. SED-based desalination pilot studies showed promising results. Ghyselbrecht et al. [166] used a SED cell exhibiting 380 cm² of effective surface area per membrane. After 105 h, they manage to double Mg^2+^ content in a 200 L North Sea water sample batch. Xu et al. [118] observed consistent desalination efficiency of brackish groundwater from bench to pilot scale (effective surface area per membrane, 3200 cm²). Recently, a modified SED stack, selectrodialysis with bipolar membrane (BMSED) has been developed to extend the applicability of this technology. Taking advantage of the ability of bipolar membranes to generate hydrogen and hydroxide ions, as well as the retention of divalent ions by monovalent permselective membranes, BMSED was successfully implemented for base/acid production [167], and lithium hydroxide production [168].

Although researchers are studying more complex effluents for specific ion recovery, SED prototypes still exhibit moderate current efficiencies for divalent ions, up to 43% for SED and 58% for BMSED [157,167,168]. A significant drawback for SED is the cost of MVCs and MVAs compared to IEMs, however Jiang et al. [169] recently developed a new way to prepare these membranes by fouling deposition. During this study, sulfonated poly (2,6-dimethyl-1,4-phenylene oxide) (SPPO) was successfully deposited on the membrane, leading to a uniform surface modification and an enhanced increase permselectivity even higher than commercial MVA. Other researchers fabricated MVAs with amphoteric materials based on an imidazolium-functionalized poly (arylene ether sulfone) and sulfonated polysulfone. The synthesized membranes exhibited low swelling ratio and high Cl^−^ selectivity [170]. Wang et al. [165] developed MVC by interfacial polymerization of a thin-film composite (TFC) on a commercial CEM to better predict the purity of final SED products. However, the stability of such membranes, as well as the full understanding of the permselectivity mechanism induced by the fouling layer or TFC layer, respectively, would require further investigations before potential use in large-scale studies or industrial implementation.

### 4.5. Electrodialysis with Polymer Inclusion Membrane (PIM-ED)

Liquid membranes consist in a layer of organic solvent in between two aqueous solutions [171]. The more common examples are supported liquid membranes (SLMs), however their poor stability hinder greatly scale-up considerations [172]. Therefore, although liquid membranes are used in ED stacks for about 50 years for metal ions recovery as an alternative to solid IEMs, practical studies remain scarce [171]. A noticeable example would be the use of an organic liquid film sandwiched between two CEMs as an electrodialytic cell for selective lithium removal [173].

In order to improve the efficiency and selectivity of liquid membranes, another alternative has been developed in the form of polymer inclusion membranes (PIMs). These membranes contain an extractant or carrier working as an ion-exchanger or a complexing agent to transport specific species across the membrane according to a concentration gradient. A base polymer is used as the structure of the membrane while a plasticizer can be added to tweak the mechanical properties of the assembly, providing more flexibility [172,174]. Contrary to SLMs which work like a compartment containing a solution, PIMs take the form of a cured composite film. Therefore, PIMs exhibit better long-term stability than SLMs and allow for a much thinner design [172,175]. PIMs are used in analytical chemistry for sample pre-treatment due to their high selectivity [176]. Although those membranes have been used for electro-membrane extraction (EME) [177], their implementation as part of an ED cell is more recent.

Electrodialysis with polymer inclusion membrane (PIM-ED) consists in using a PIM to replace a specific IEM of a conventional ED stack. The diluate compartment is called the feeding solution while the concentrate compartment is the stripping solution [178]. The PIM provides a lower electric resistance to promote ionic transport as well as an enhanced selectivity for specific ions. When aiming to transport anions, an overwhelming advantage of those membranes is the blockage of hydrogen back diffusion compared to AEMs for which proton leakage is often reported [179]. The latest PIM-ED applications used ionic liquids as the extractant, typically, quaternary ammonium and quaternary phosphonium salts [175,178,179]. Alternatively, Hoshino [180] used N-methyl-N-propylpiperidium bis (trifluoromethanesulfonyl) imide (PP13-TFSI) as the ionic liquid to impregnate organic membranes and achieve a selective concentration of lithium from seawater. Other PIM-ED applications include the separation of volatile fatty acids, acetic and hexanoic acids [175] and the removal of Cr (VI) as HCrO_4_^−^ [178,181].

### 4.6. Electro-Electrodialysis (EED)

**Figure 18:**
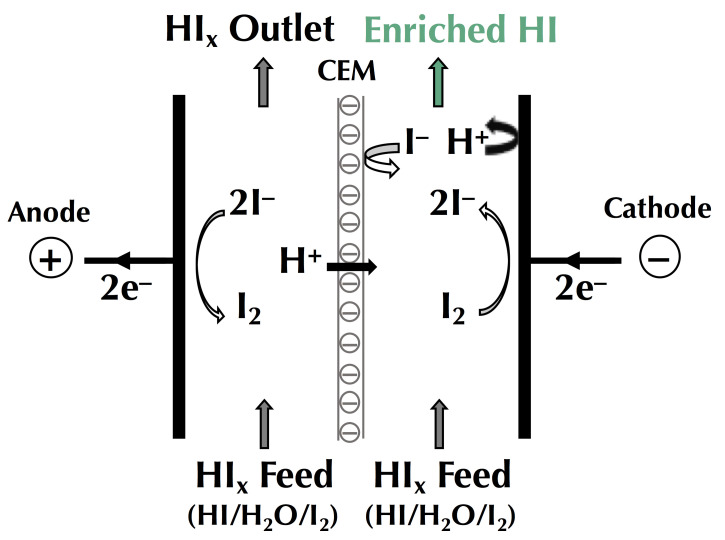
Principle of electro-electrodialysis applied to iodine-sulfur process for hydrogen iodide concentration. CEM: cation-exchange membrane.

Based on an ED stack with generally only two compartments separated by an IEM to allow selective ionic migration, electro-electrodialysis (EED) also relies on electrolysis. By generating redox species at the electrodes, it is then possible to further modulate the products obtained in the recovery compartment. Traditional EED applications include organic/inorganic acid purification and recovery as well as hydrogen iodide (HI) concentration for hydrogen production [4,128,182,183]. Although the idea of using EED as part of the thermochemical water-splitting process for hydrogen production goes back to the early 1980s [183], modern interest for renewable and sustainable energies led researchers to constantly innovate in order to improve this process [184]. Additionally called the sulfur-iodine thermochemical cycle, the process uses iodine (I_2_), sulfur dioxide and water as reactants. It primarily consists of sulfuric acid and HI production by “Bunsen reaction”. A following “sulfuric section” allows for the regeneration of sulfur dioxide and water. While “HI_x_ section”, the most critical step in terms of efficiency and energy consumption, consists of the concentration of HI followed by its thermal decomposition in regenerated I_2_ and dihydrogen (H_2_) as the final product [185]. EED equipped with a CEM has proven to be an effective technique for the concentration of HI as represented in Figure 18.

The current impetus towards EED can also be explained by recent breakthroughs regarding membrane materials. Although CEMs are produced reliably at commercial scale [186], new materials are still investigated to improve H^+^ selectivity and overall performances of the EED systems. Tanaka et al. [187] studied the performance of EED for HI concentration using a CEM made from poly (ethylene-co-tetrafluoroethylene)-styrene grafted polymer (ETFE-St) and a commercially available Nafion 212 CEM. They found that the H^+^ selectivity was mostly influenced by the back diffusion of I^−^ which depended on the affinity of the membrane material with I_2_. An acid and oxidative resistant sulphonated copolymer (PVSU) synthesized by chemical grafting of 2-methyl-2-(N-(3-sulfopropyl) acrylamido) propane-1-sulfonic acid (MSAPS) with dehydrofluorinated poly (vinylidene fluoride-cohexafluoropropylene) (PVDF-co-HFP) was also recently studied as CEM material [188]. While PVSU CEMs allowed a near 100%-current efficiency, it was shown that the high concentration of fixed charges in the membrane matrices significantly reduced the energy consumption required to produce 1 mol H_2_. The stability of AEMs, mainly used for organic and inorganic acid purification, is a significantly higher hurdle compared to CEMs [182]. The reasons for it are the chloromethylation and quaternization steps during AEM production process which remain technically challenging [186]. As such Duan et al. [186,189] proposed an alternative way to synthesize new polysulfone-based membrane materials: quaternary ammonium polysulfone (QAPSU) and comb-shaped quaternized ammonium polysulfone (Cx-QAPSU). Using chlorotrimethylsilane as a non-toxic chloromethylation reagent and trimethylamine ethanol solution as the quaternization reagent allowed for an efficient synthesis. QAPSU and Cx-QAPSU used in an EED stack led to 65% and up to 85% metal ion removal, respectively, as well as higher current efficiency and lower energy consumption for phosphoric acid purification than commercial LE 1201 membrane [186,189].

Other applications of EED were recently developed to answer current environmental issues regarding water contamination. Wu et al. [190] studied the feasibility of EED for phenol removal from salty wastewater. The process is based on hydroxide generation at the cathode, where phenol could be converted in phenoxide ions which, after transport through the AEM, could be converted back as phenol molecules in the anode compartment. Although 90% removal of phenol was achieved, it was found that hydroxide and sulfate anions are competing with phenoxide anions leading to an increased energy consumption. Ideal conditions were obtained at high phenol and low salt concentrations in the feed [190]. Similarly as SED, EED was implemented with bipolar membranes (EEDBM) for lithium recovery from artificial lake brines [191]. In comparison to conventional EED or EDBM it allowed to produce lithium hydroxide both in the base and cathode compartments leading to an increased current efficiency.

### 4.7. Membrane Capacitive Deionization (MCDI)

Capacitive Deionization (CDI) is a non-membrane technique first described in the 1960s and since then mainly used for water desalination. It differs from conventional ED as it is based on electrode reactions to remove and concentrate ions. A phenomenon called electrosorption consists in the electrostatic attraction and adsorption of ions at the interface between the solution and the porous electrodes while an electric potential is applied. Then, ions can be desorbed, thus regenerating the electrodes [4,192,193]. The adsorption capacity of the electrodes is considered to be the most important factor in terms of performances [193]. A more recent technology derived from CDI by adding IEMs in order to improve its results was incepted in the mid-2000s [194]: membrane capacitive deionization (MCDI). Membranes may seem of secondary importance compared to electrodes; however, they have the key role of improving the charge efficiency by avoiding the transport of co-ions of similar charge as the electrodes. Furthermore, MVC or MVA can be used to optimize experiments with complex multi-ion solutions. It is also possible to coat the membrane directly on the electrode, allowing a thinner layer than a separate membrane, however long-term performances are still to be evaluated [195].

**Figure 19:**
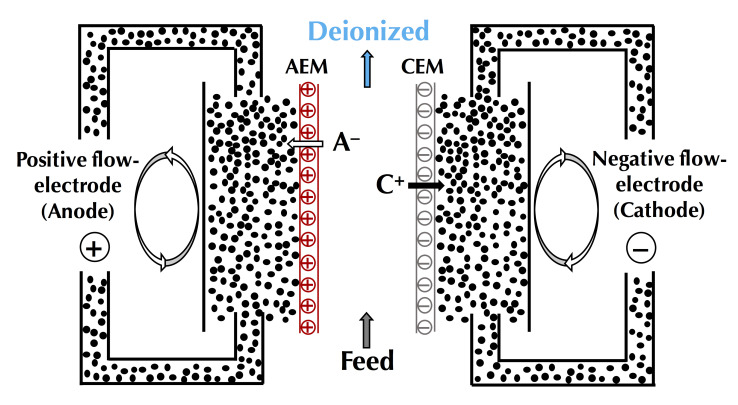
Principle of flow-electrode capacitive deionization. CEM: cation-exchange membrane; AEM: anion-exchange membrane; A^−^: anions; C^+^: cations.

Flow-electrode capacitive deionization (FCDI) is the most recent and promising MCDI-derivated technologies (Figure 19). The use of membranes in CDI helped develop the concept of flow-electrode, where active carbon particles circulate in an electrode compartment separated from the feed stream by IEMs [195,196]. Contrary to stationary electrodes which require a distinct regeneration/desorption step, having a flow electrode enables a continuous operating mode as carbon particles are constantly recycled which improve duration, effective ion removal and energy consumption of the process [197,198]. It is also possible to process higher salinity streams than when using static porous carbon electrodes [195,198]. Latest advances showed that it is possible to further improve the overall cell conductivity and charge efficiency by working in overlimiting conditions and thus bolstering electroconvection [199]. Liang et al. [200] also enhanced the charge efficiency by adding carbon black as a conductive agent in the flow-electrode compartments. In another study, AEM-electrode assembly was found to reduce potential drop and provide a more homogeneous electric field [201]. Ma et al. [202] investigated the stacking of multiple membrane pairs in a FCDI cell and demonstrated the feasibility of semi-continuous operation with two membrane pairs. However, hurdles regarding scale-up considerations are still high, especially the possibility of water transfer through the IEMs.

Although CDI municipal waste water desalination units exists at large scale (e.g., more than 30 industrial systems in China with 100–2000 m³/h capacities), MCDI is still struggling for implementation at industrial scale. Membrane cost and performances are critical factors. In addition to recent FCDI developments, Suss et al. [195] suggested that the development of PIM adapted to MCDI could be a decisive step forward as it would help prevent leakage of unwanted ionic species and water.

### 4.8. Continuous Electrodeionization (CEDI)

**Figure 20:**
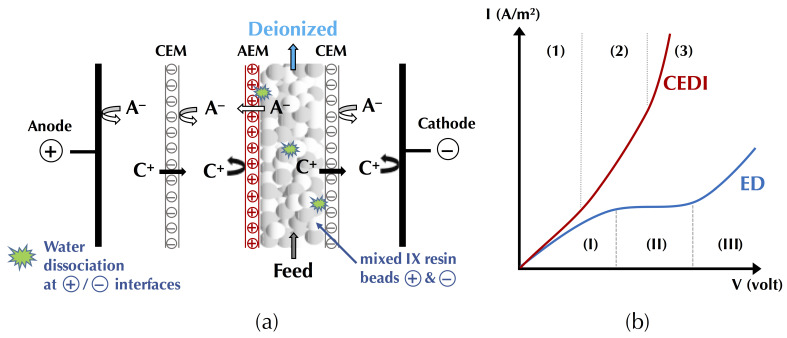
(**a**) Principle of continuous EDI (CEDI) and (**b**) current regimes in CEDI and ED (adapted from Hakim et al. [205]). CEM: cation-exchange membrane; AEM: anion-exchange membrane; IX: ion-exchange; A^−^: anions; C^+^: cations.

Called electrochemical deionization, electrodeionization (EDI) or conductive electrodialysis (CED), this particular variant of ED appeared in the 1950s and is now considered as a next-generation technology and a green process for water purification such as deionized water [203,204]. Its development was relatively slow due to the difficulty to grasp its kinetics mechanisms. EDI is based on an electrodialytic cell which use ion-exchange (IX) resin to improve ion flux through the membranes and conductivity as display in Figure 20a [205]. Resin beads can either be placed in the dilute compartment or both dilute and concentrate. Moreover, anion-exchange resin and cation-exchange resin beads can be placed in separate compartments or in the same one leading to mixed IX resin beads [206,207]. The EDI process can be summed up in 2 main steps: the diffusion, in the dilute, of cations and anions through the anion-exchange resin and cation-exchange resin beads, respectively, corresponding to the IX step, then, after ionic diffusion from the beads to the membrane, ions are migrated through the membrane, under the effect of the electric field, which corresponds to the ED step [4,208]. Compared to conventional ED, EDI has a lower electrical resistance allowing to process low concentration streams [205]. And while ion mobility is about 20 times greater in solution than within the solid IX resin, the ionogenic sites of the resin can gather 1000–100,000 times more ions than found in solution. Therefore, as transport rate is also determined by concentration (in addition to ion mobility), mass transport by successive collections/discharges in the IX resin can be 50–5000 times faster than in solution [203].

The ingenuity of CEDI is that contrary to batch mode EDI, the IX resin can be regenerated continuously in situ in overlimiting conditions due to water splitting in H^+^ and OH^−^ at the interface of anionic and cationic exchange materials (beads, membranes) (Figure 20a) [203]. This feature is one of the main evolution compared to traditional EDI and more advanced forms of IX such as mixed-bed ion-exchange (MBIX) [209], also known as mixed-bead Deionization (MBDI) [192], which uses strong mineral acids and bases for the regeneration of the resin, greatly lowering environmental-friendly aspects. This continuous feature as well as the high conductivity of the cell allow for a deep deionization compared to ED and IX taken individually. CEDI is then sometimes referred to as hybrid ion exchange/electrodialysis (IXED) [207]. The first commercial inception of CEDI was in 1987 and about three decades later, thousands of commercial CEDI systems produce high purity deionized water with 0.1–1500 m³/h operating capacities [204,206]. Electrical consumption for water treatment by CEDI is usually 0.05–0.8 kWh/m³ depending on effluent and purity targeted. The main parameters responsible for its efficiency are the current strength, the flow velocity, the temperature and the total dissolved solids in the compartments [206].

Recent advances based on a better understanding of kinetics mechanisms allowed to explore other utilizations such as fractional electrodeionization (FEDI^®^), recently implemented at large scale. Bolstered by a 60-stack plant with a 105-m³/h capacity, this water-purification technology is already well-established and can produce highly purified water [210,211]. It is operated following two distinct stages of EDI. During the first stage, a low voltage is applied to the system in order to remove strongly charged species. As hydrogen cations are more mobile than hydroxide anions, they are preferentially transported towards the concentrate compartment where pH 4.5–5 can be obtained, thus limiting scaling caused by multivalent cations. The second stage at higher voltage is taking place at a higher pH (9–9.5) due to the excess of hydroxide anions in the feed compartment which allows a deeper deionization of weakly charged silica ions. Using two stages improves the flexibility of the technology, allowing a larger range of hardness in the feed [205,211]. Other technological achievements include electrodeionization reversal (EDIR) with polarity inversion to reduce fouling [212,213] or electrodeionization with bipolar membranes (EDI-BM or BMEDI) in order to regenerate the IX resin with H^+^ and OH^−^ ions produced by the bipolar membrane [207]. Furthermore, the development of high-performance materials fostered wafer enhanced electrodeionization (WE-EDI) or resin wafer EDI (RW-EDI) for which IX resin is combined with a binding agent to reduce issues of ion leakage and uneven flow distribution. By using a water-dissociation catalyst, Jordan et al. [214] developed a new bipolar ion-exchange resin wafer to promote ion conductivity. Other materials such as ion exchange textile led to improved current efficiencies 1.5 times higher than conventional ED [205].

The comprehension of electrodeionization overlimiting behaviours should promote the next advances in the field. Water-splitting and electroconvection in CEDI exhibit different behaviours compared to overlimiting dynamics observed in conventional ED [208]. In CEDI, there is no distinct plateau in the limiting current regime as opposed to what is observed in ED (see Section 3.3.2). The three regimes in CEDI reflect the increasing conductivity of the IX resin: the transport region (1), the water dissociation region with a higher V–I slope (2) and the limiting current region with an even steeper V–I slope (3) (Figure 20b) [205]. Park and Kwak [208] established that even though different slopes V–I are possible, a unique characteristic of overlimiting CEDI, demonstrating the occurrence of electroconvection, is the sudden increase in the standard deviation when measuring the current multiple times. It differs from the water-splitting region where no substantial current fluctuations are noticeable. When placed at the limit between the two overlimiting zones, microscopic observations of CEDI flow channels showed a synergetic effect of water-splitting and electroconvection [208]. The resulting optimal current efficiency and energy consumption provide a solid basis for further implementation of overlimiting CEDI.

### 4.9. Shock Electrodialysis (Shock ED)

**Figure 21:**
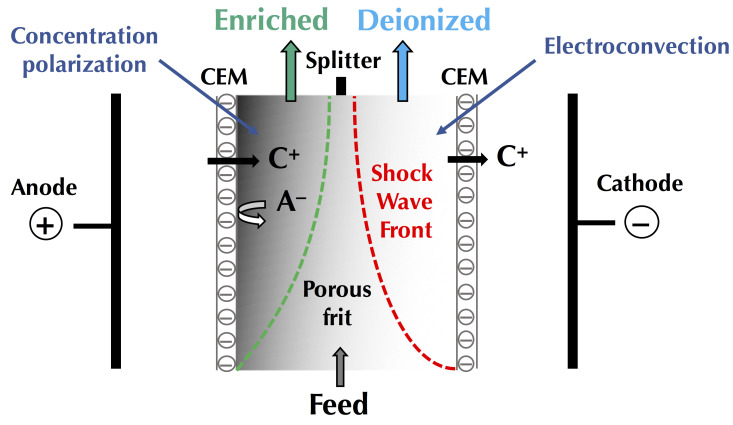
Principle of shock ED. CEM: cation-exchange membrane; A^−^: anions; C^+^: cations.

The shock electrodialysis (shock ED, sometimes also referred to as SED but not to be confused with selectrodialysis) technology has been developed in the early 2010s [215], following the mathematical and experimental evidence of “deionization shocks” [216,217,218]. The deionization shocks can be conceptualized as sharp concentration gradient waves occurring when applying overlimiting current in a weakly charged porous media, sometimes referred as to “leaky membranes” [215]. This discovery lead to the patenting of a desalination and purification process [219]. The principle of shock ED is represented in Figure 21. In overlimiting current conditions, a depletion area is formed following the progression of a concentrated deionization shock front. Furthermore, electroconvection is promoted which allows cations to rapidly be transported through the CEM on the cathodic side. Close to the CEM on the anodic side, the propagation of the shock front leads to the formation of a concentration polarization area consisting of cations transported from the anodic compartment and anions rejected by the CEM. By using a physical splitter, it is then possible to segregate both area into an ion-enriched and a deionized product stream [220,221,222].

The marketing strategy behind shock ED is rather unexpected but compelling. According to Schlumpberger et al. [220], their small-scale prototype is “scalable”, although not yet able to compete with large-scale RO or ED systems. Therefore, they suggest that it should be developed as a portable strategy for emergency specific use in case of natural disasters for example or in remote areas. Current equipment could produce 20 L of purified water per day [220]. It is an interesting and readily available alternative to the usual approach as it skips the extensive steps towards industrial scale-up Another novel feature brought by shock ED is the combination with functionalities other than deionization. By tweaking the nature of the porous media, shock ED can exhibit micron-scale and nanoparticle aggregates filtration as well as bacteria elimination either by filtration or intense electric field [223].

Latest developments of shock ED focussed on diversifying the potential applications as well as optimizing performances. Alkhadra et al. [221] developed a small-scale unit for decentralized desalination using sodium citrate buffer as a cleaner electrolyte and obtained a 99.98% rejection of salts. A similar shock ED configuration was studied and selectively removes divalent magnesium cations (>99% Mg^2+^) due to different mobility mechanism compared to monovalent Na^+^ [224]. This technology has also been implemented as a multi-step process to remove radionuclides from artificial contaminated water simulating effluent from nuclear energy production. After three recirculations through the shock ED system the authors were able to remove 96.3% of Cs^+^, 97.6% of Li^+^ and 99.6% of Co^2+^, showing promises as a new method for radioactive wastewaters treatment [225].

### 4.10. Electrodialysis with Filtration Membrane (EDFM)

**Figure 22:**
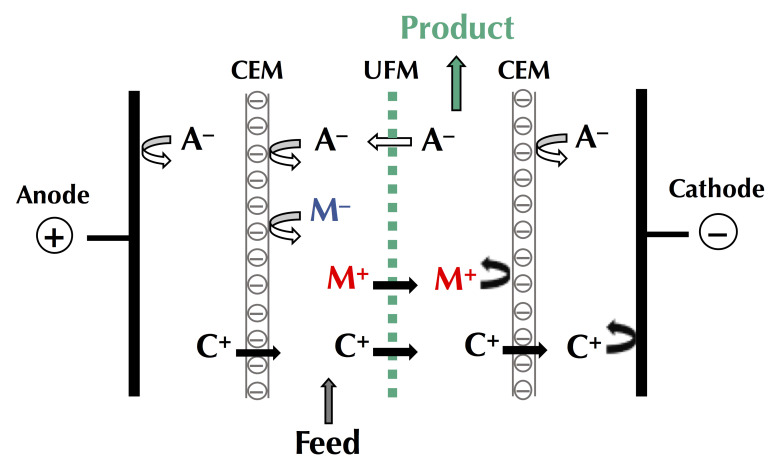
Principle of EDFM using a cationic configuration. CEM: cation-exchange membrane; FM: filtration membrane, A^−^: anions; C^+^: cations; M^−^, M^+^: charged macromolecules.

Electrodialysis with filtration membrane (EDFM) is gaining an increasing interest as a selective separation process and was recently reviewed thoroughly by Dlask and Václavíková [226] and Sun et al. [227]. It involves the integration of filtration membranes to the electrodialysis stack, mainly ultrafiltration or nanofiltration membranes (Figure 22). In the next sections, the principles and latest development of this technique are presented.

#### 4.10.1. Electrodialysis with Ultrafiltration Membrane (EDUF)

While Galier and Roux-de Balmann [228] investigated the integration of ultrafiltration membranes (UFMs) in electrophoretic membrane contactors, Bazinet’s team developed a new type of electrodialytic configuration by using FMs to replace IEMs in order to broaden the range of ED applications. Exceeding the 500-Da molecular weight cut-off (MWCO) of IEMs, UFMs could allow the migration of charged species of higher molecular weights. After the study of the EDUF (electrodialysis with ultrafiltration membranes) process for the purification of green tea flavonoids [229], the separation of bioactive peptides [230] and by optimizing the performance of UFMs [231], Bazinet et al. [232] patented the technology. An EDUF cell consists in the stacking of IEMS and UFMs as represented in Figure 22. In this example, positively charged macromolecules selectively migrate from the feed compartment toward the recovery compartment. However, by using an inverted configuration with switched feed and recovery compartments, negative macromolecules could be instead recovered. An improved configuration was developed based on two recovery compartments surrounding the feed compartment [233]. This way, both positive and negative macromolecules could be retrieved. This configuration has been used to selectively separate positive and negative peptides contained in hydrolysates derived from vegetal [234] and animal [235,236] by-products. A limitation of the technique is water transfer. Osmosis phenomena is susceptible to occur due to the difference in concentration between the feed and product compartments, water can then be transferred through the membrane [237]. Although limited in ED, it can be expected in larger proportion in EDUF due to the higher permeability of UFMs compared to IEMs. However, the studies of such phenomenon would need to be supplemented.

Recent EDUF advances include the stacking of UFMs with different MWCOs in order to improve size-selectivity as well as the in-situ enzymatic hydrolysis. Hence, the recovery of 4 fractions with two cationic compartments and two anionic compartments was investigated [238,239]. Furthermore, triple selectivity was obtained by stacking three UFMs of decreasing MWCOs [240]. Cationic and anionic configurations were tested. The evaluation of the different fractions in terms of glucose uptake led to the identification of 11 peptides with antidiabetic potential. Interestingly, the lowest-MWCO cationic compartment contained inhibitor peptides and amino acids, while the bioactive peptides were concentrated in the highest-MWCO cationic compartment. To avoid the pre-hydrolysis of the protein before EDUF treatment, Doyen et al. [241] and more recently Suwal et al. [242] tested the simultaneous enzymatic hydrolysis and fractionation of generated bioactive peptide. They observed that the peptide sequence ALPMHIR, identified as lactokinin and known to exert an important antihypertensive effect, was recovered with an estimated 66% migration rate [241] and that the peptide migration rate was found to be affected by the mode of enzymatic hydrolysis and separation [242]. Current research is exploring other ways to optimize hydrolysis before EDUF, e.g., by using high hydrostatic pressure [243]. Recently, Wang et al. [244] synthesized a polyvinylalcohol (PVA) membrane aiming at a 150-kDa MWCO for use as a filtration membrane in an EDUF stack. They experimented on artificial milk in order to selectively recover glycoproteins (lactoferrin and immunoglobulins) and observed promising concentration in the retentate compartment compared to other dairy proteins tested. An alternative EDUF configuration was explored by Tamersit et al. [245] for the desalination of tannery wastewaters. By protecting the AEM with an UFM in the electrodialytic cell, they managed to totally prevent peptide and protein fouling. Pulsed electric fields and polarity reversal were also studied in EDUF as a way to prevent fouling [105]. Both modes reduced significantly fouling, especially on AEMs, while PEF (2 s pulse/0.5 s pause) provided an increased selectivity for arginine and lysine-based peptides compared to direct current mode. In 2020, predictive models were developed for determination of peptide fouling [246] and peptide migration/selectivity [247] based on the physicochemical characteristics of the filtration membranes (conductivity, contact angle, % of hydrophilic pores, porosity, zeta-potential, Arithmetic mean of roughness Ra and Maximum height Rz), their pore size (from 5 kDa to 300 kDa) and their material (PES, PAN, PVDF and PVC-silica). The mechanisms involved in the fouling of peptide and their migration were also deeply studied as a function of these characteristics [246,247].

The recent breakthrough regarding PEF applied to ED technologies should promote their future investigation in EDUF for low-cost and efficient processes. In the same vein, although a preliminary study was carried out regarding overlimiting conditions in EDUF [248], additional exploration of electroconvection in EDUF would benefit to the field. The scale-up of this technology at a pre-industrial scale, in collaboration with an equipment manufacturer, is currently underway in our team for bioactive peptide separation.

#### 4.10.2. Electrodialysis with Nanofiltration Membrane (EDNF)

The use of charged filtration membranes stacked in an ED cell was demonstrated to be effective for demineralization of solutions. Hence, in 2011, Bazinet and Moalic were the first to use a nanofiltration membrane (NF) in an ED stack for the demineralization of sea water [249]. They demonstrated that the use of NF membrane can also allow the selective separation of cations. More recently, Ge et al. exemplified such a selective cation fractionation of EDNF with H^+^/Zn^2+^ and Na^+^/Mg^2+^ systems and demonstrated that an NF membrane can increase the limited current density in ED [250]. In 2020, EDNF was tested for acid whey demineralization and simultaneous lactate recovery in under- and over-limiting conditions [90]. The integration of a nanofiltration (NF) membrane in an ED conventional stack led to a similar lactate recovery rate than conventional ED (around 29% in 60 min) with respective demineralization rates of 20.3% and 50.8%. In overlimiting conditions, around 36% lactate recovery were obtained for both configurations, in 30 min, but with demineralization rates of 77.2 and 27.5% for conventional ED and EDNF respectively. However, for the EDNF configuration, the recovery of lactic acid was almost doubled in the overlimiting condition. Different hypotheses were proposed to explain these results; (1) the pore size of the NF membrane since NF membranes have a high permeability for monovalent cations and organic acid anions with low molecular weight, but they limit the passage of organic compounds with a molecular mass that exceed 300 Da (ex. lactose) [251,252], (2) the temperature evolution during the process because when the system reaches higher temperature, there is expansion of the polymer included in their structure [253] and (3) the fact that no LCD was obtained for NF membrane, since H^+^ produced, in the whey compartment at the interface of the AEM after the LCD was reached would have reacted with some lactate anions (pKa of 3.86) to produce non-charged lactic acid, decreasing their potential to migrate. Concerning the relative energy consumption (REC), in underlimiting conditions no difference in REC, for demineralization (around 20 Wh/g K^+^) and lactate recovery (around 8.6 Wh/g lactate) steps, for both membrane configurations was reported. However, when the underlimiting current condition was applied similar REC were calculated for the demineralization step (around 163 Wh/g K^+^) but around 68% more energy was needed for conventional ED (109.5 vs. 65.1 Wh/g lactate): the overlimiting condition increased by 7–9 times the energy consumption compared to the underlimiting condition [90]. Recently, Ye et al. [254] used EDNF for textile wastewaters treatment. Their novel loose NF-based ED process, exhibited high permeation of salt (98.9% desalination) while retaining dyes (99.4% recovery) with low fouling showing promises as a sustainable depollution process.

### 4.11. Reverse Electrodialytic Technologies

**Figure 23:**
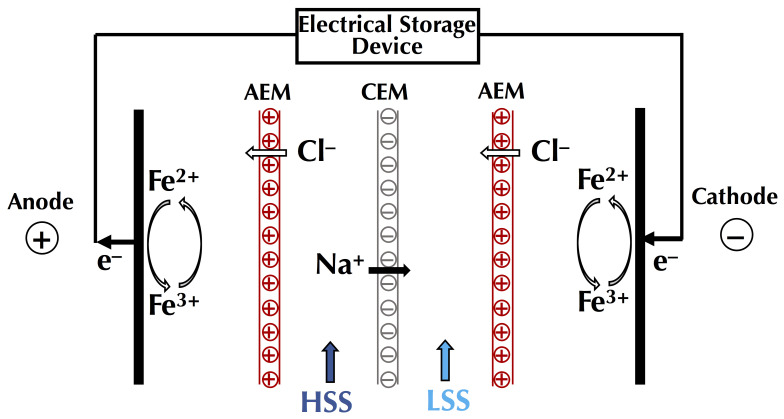
Principle of reverse ED. CEM: cation-exchange membrane; AEM: anion-exchange membrane; HSS: high-salinity stream; LSS: low-salinity stream.

In a context of depletion of natural resources, reverse technologies are promising alternatives for clean energy production. Reverse electrodialysis (RED) is based on salinity gradient power (SGP) also called “Blue Energy” which is thought to represent 2.4 TW when considering estuaries worldwide, i.e., 80% of 2020′s global electricity generation [255]. By using ionic concentration gradients, RED promotes the flow of ions in a non-powered ED stack in order to generate electric energy. As displayed in Figure 23, high-salinity stream (HSS) and low-salinity stream (LSS) are used as feed solutions. Their adjacent compartments are separated by IEMs, according to a conventional ED configuration. The concentration gradient between the two compartments induces anion and cation migration from the HSS towards the LSS selectively across the AEM and CEM, respectively. Electrode solution consists of a redox rinse, usually FeCl_3_ and FeCl_2_. As such, the transport of Cl^−^ to the electrolyte at the anode triggers an oxidation of the Fe^2+^/Fe^3+^ couple, while the transport of Cl^−^ from the cathodic electrolyte leads to a reduction, the resulting exchange of electrons generate electric power [256,257].

Similarly to other electrodialytic processes, RED’s early inception (1954) led to slow developments correlated with the inadequate performances of membrane materials at this time. Current membranes exhibit higher conductivity and improved selectivity allowing to reach power densities in the range of 2–10 W/m² at lab scale [258,259]. Flow dynamics have a substantial impact on power density results leading to the development of turbulence-inducing woven spacers [260,261]. Furthermore, membrane permselectivity has proven to be another critical factor for RED, since divalent cations can greatly affect the power capacity causing an efficiency drop and increasing the membrane resistance by possible scaling [261,262,263]. The development of monovalent permselective membranes based on charge repulsion or steric exclusion as well as the better understanding of scaling mechanisms, especially in relation to the pH, paves the way toward improved more viable performances [85,263,264,265,266]. Yet, RED applications on close systems relying on artificially-prepared streams of monovalent ions still outperform by far equipment implemented in natural environments such as estuaries for which organic fouling also must be considered [257,267,268,269]. Due to the permselectivity, scaling and fouling issues, when looking at implementations in “real” conditions, power densities range from –0.01 to 0.65 W/m² for treatments of 1–3 months [265]. Hence, Tedesco et al. [264] studied the performances of REAPower, a pioneer RED pilot plant in Italy with a 1 kW overall plant capacity objective which demonstrated 700 W with artificial NaCl streams vs. 330 W with natural brine and brackish water. Bolstered by 500 cell pairs for a total of about 100 m², the plant achieved a power density of about 0.8 W/m² over 5 months with real streams [259]. Another pilot plant was established in The Netherlands (Blue Energy project) aiming for an overall capacity of 50 kW while the next stage would be a demo pilot up to 2 MW [270]. More recently, a pilot plant in South Korea exhibiting 1000 cell pairs for a total membrane surface of 250 m² managed a power density of 0.38 W/m² using seawater and wastewaters [262]. Gómez-Coma et al. [271] implemented a 20-day RED experiment with power densities up to 1.4 W/m². Although carried out with a small-scale RED, natural sea water and reclaimed water from wastewater treatment plants were used as feeds with no fouling. Despite these encouraging results, the economic viability of RED is still precarious, largely due to membrane cost and maintenance as well as the low current densities attained. Power densities greater than 2 W/m² were evaluated as economically suitable for capturing SGP [263,272]. However, more profitable strategies are currently studied by combining RED with sea water desalination units for better eco-efficiency (see Section 5.1.3). In addition, efforts are being made to develop new reverse electrodialytic ways to produce and store SGP [257].

Even though they provide better flux dynamics by promoting turbulence, spacers are known to be more responsible for organic fouling issues than membranes [269]. RED with membrane-electrode assembly (MEA) was developed as a non-spacer technology to lower compartment resistance and improve power density. Compared to conventional RED using similar NaCl streams, MEA-based RED at small scale attained higher power densities (up to 4.1 W/m²) [255]. Capacitive reverse electrodialysis (CRED) is another alternative of RED technology [273]. Similarly to MCDI, it stages capacitive electrodes of active carbon. Those generate electricity without redox reaction (about 1 W/m² with small-scale 30-cell equipment), thus preventing the use of specific chemicals. However, electrodes would still need to be regenerated [274]. Similar average current densities were achieved by a capacitive concentration flow cell using CEMs and carbonized peat moss electrodes [275]. RED with flow electrodes (FE-RED) was developed to allow continuous operation by recirculating the electrodes. Its huge advantage is a constant electricity generation, however current productivity is limited due to maximum power densities not exceeding 0.26 W/m² [276]. Other technological developments include reverse electrodeionization (REDI) showing a decreased system resistance compared to RED due to embedded IX resin in the dilute compartment, leading to 0.32–0.44 W/m² power densities [277,278]. Reverse electro-electrodialysis (REED) was also developed to mitigate RED’s shortcomings and produce hydrogen for energy. Chen et al. [279] experimented on a REED stack using strong acid/base as electrolyte to boost electrochemical performances, in particular reduction of H^+^ in gaseous H_2_ at the cathode. They attained a 0.45-W/m² power density and a H_2_ production rate of 0.55 mL·h^−1^·cm^−2^ electrode, but only 2% hydrogen energy recovery due to small scale limitations. RED with bipolar membranes are currently studied for potentially higher power densities [280]. Finally, a new nanoporous carbon membrane with mesoscopic thickness have been developed for reverse electrodialytic applications and reached a maximum power density of 67 W/m² [281]. Despite showing increasing promises for SGP energy storage, these recent developments are still in need of further studies establishing larger-scale practicality.

## 5. Integration of ED Technologies in New Sustainable Strategies

Current ED processes are making a growing contribution toward sustainable pure water production. Moreover, these are very promising for energy, wastewaters remediation, fine chemicals and food applications. In this part, we present recent intensification developments and strategies involving new and mature technologies, and discuss their eco-efficient potential. Eco-efficiency is a management tool that relates life cycle environmental indicators and product value to support decision making [282,283]. It aims at identifying scenarios that decrease environmental impact while increasing the product value. Indeed, to improve the eco-efficiency, one can choose a different supplier, change to a greener process or technology, or develop a product with a stronger valued market [284].

### 5.1. Desalination and Salinity Gradient Power Recovery

#### 5.1.1. Desalination Strategies for Ultrapure Water

Desalination of sea water using conventional ED is a robust process with moderate energy requirement. ED represents 4% of the desalination capacity worldwide, while reverse osmosis (RO) stands for 64% and thermal distillation processes for about 30%. In addition to a more efficient energy use than for thermal processes, ED has several advantages compared to RO. Lighter pre- and post-treatments lead to longer membrane lifespan, while higher temperature tolerance and selectivity allow more flexibility. ED has also better water recovery [6,285]. In terms of cost effectiveness, ED is generally better for water with less than 5 g of total dissolved solids (TDS) per liter, while RO is more interesting for streams with concentrations higher than 5 g TDS/L [192]. Due to this complementarity and in order to reach deeper desalinations by maintaining the eco-efficiency of the process, researchers are studying the coupling of electrodialytic technologies with other operation units such as pressure-driven membrane filtration.

Continuous electrodeionization leads to higher water purity than ED or RO due its enhanced conductivity (c.f. Section 4.8), however it requires less concentrated water as the feed stream (<30 ppm). Therefore, a viable strategy is to associate CEDI with RO, ED or both [205]. Ho and Wood [286] evaluated the performances of a full-scale RO/CEDI plant for deionized water production. After 18 months, 3.4 million m³ of ultra-pure water (conductivity < 0.1 µS/cm i.e., resistivity > 10 Ω.cm) was produced. More recently, a high efficiency reverse osmosis (HERO™)/FEDI^®^ process has been implemented in Egypt as a water purification strategy [210]. Nile water and various effluents were successfully converted in ultra-pure water with resistivity ranging between 10 and18 MΩ.cm. Water discharged by geothermal plants is gaining more and more interest as a sustainable source for fresh water [206,287] so current strategies involve RO applied geothermal water followed by CEDI. Although such processes were originally aiming for ultra-pure water (0.05–0.10 µS/cm), recent implementations only manage to reach purified water grade (2–50 µS/cm), which still stands for a high-value product [288]. MCDI was also considered in combination with RO. However, electrode lifespan of MCDI is a critical drawback in the long run when aiming for eco-efficient strategies. The superiority of ED compared to MCDI in terms of energy cost to reach deeper desalination and fouling/scaling control associated to smaller equipment explains why the former is usually preferred for coupling with RO [289]. To mitigate these issues, Chung et al. [197] studied a small-scale RO-FCDI process for sea water desalination. The batch-mode implementation of FCDI in replacement of a second RO pass led to 95% removal while energy consumption (1.3 kWh/m³) was three-times higher than full-scale RO (<0.4 kWh/m³). However, according to the authors’ projections, this difference would be mitigated by scaling-up the technology.

#### 5.1.2. Lower-Grade Fresh Water

Drinking water (and even more so, irrigation water) does not require as much ion removal as ultra-pure water used in pharmaceutical, chemical or semi-conductor industries, leading to strategies yielding lower added value but associated to easier implementation. Aiming for zero liquid discharge (ZLD) as part of a fresh water production strategy, Chen et al. [153] studied electrodialysis metathesis (EDM) as a way of concentrating sea water (SW) RO reject brine before low-energy evaporation to recover solid salts, which would otherwise remain as polluting liquid waste. They managed concentration of the brine without cation-induced scaling due to the conversion of problematic low-soluble species into high solubility liquid salts. A previous pilot-scale evaluation of a similar ZLD strategy established that EDM demonstrates better eco-efficiency than thermal distillation for concentrates of 5000–10,000 ppm TDS or less [290]. Other large-scale investigations included RO and NF in their zero discharge desalination (ZDD) strategy applied to ground water. A full-scale plant was able to provide drinkable water with an estimated energy consumption of 2.3 kWh/m³ [291].

RO-EDBM is another strategy to soften water while self-supplying acid and base for maintenance purposes (membrane conditioning, scaling prevention…). Herrero-Gonzalez et al. [292] recently evaluated its environmental sustainability by life cycle assessment (LCA). They showed that the self-supply in chemicals is overshadowed by the increase in energy consumption from coupling both unit operations. The eco-efficiency of the process is then largely depending on the proportion of renewable energy in the grid mix. Taking an original approach, Lejarazu-Larrañaga et al. [293,294] studied the possibility of recycling spent RO membranes into IEMs to be used in ED for drinkable water production. They successfully implemented acid/base activation treatment to approach water fluxes and current efficiencies achieved in ED with commercial membranes, while energy consumption remained higher [294].

Displaying an opposite mode of operation to RO, forward osmosis (FO) recently gained interest being one of the lowest energy-consumption desalination process [295]. It requires an HSS called “draw” that strip water from a lower-concentration feed using osmotic gradient. Subramanian recently patented its coupling with EED as a hybrid FO-EED process for treatment of very high TDS brines (>35,000 ppm) that could not be concentrated other than by using thermal distillation. By using such strategy, he claims a final TDS concentration up to 350,000 ppm as one output, while recovering fresh water usable as drinking, irrigation or industrial water after minimal additional treatment, e.g., UV. The integration of recent advances in membrane-electrode assembly (MEA) are a supplementary option to improve performances [296]. MCDI was investigated for irrigation water production using brackish water. By coupling farm scale economic data from Australia and single-cell MCDI performance reports, Bales et al. [297] developed a model to evaluate the profitability of the approach. They estimated a final cost < 1 AU$/kL for water of different salinities depending of the crops. Further real-life experiments would confirm these encouraging results and clarify the sustainability of the strategy. Other alternatives for irrigation water production include FO-ED hybrid systems due to the low power consumption inherent to FO [298]. Most of those approaches are based on wastewater and will be developed in Section 5.2.1.

#### 5.1.3. Integration of Energy Production from Salinity Gradient Power

As mentioned in Section 4.11, engineers and researchers are still struggling to produce electricity using reverse ED technologies in an economically viable way. Although, simple filtration pre-treatments are implemented, using natural streams at large scale is requiring more extensive steps in order to reach performance levels obtained at smaller scale with artificial NaCl solutions. One strategy consists in increasing the profitability of the process by including water purification as another output. This synergistic approach recently called power-free electrodialysis (PFED) [299] was implemented on several small-scale studies (<200 cm² effective surface area per membrane) [6,257]. Chen et al. [300] brought back this concept and adapted it for application in an insular environment. An integrated RED-ED stack was used with artificial NaCl brines. The idea of using only one set of electrodes for both types of ED cell did not allow energy recovery for other uses, however it provided an ionic current loop self-powering the system. In order to optimize eco-efficiency, a next step would be to reuse streams generated by one type of ED cell into the other. By doing so, it would limit brine discharge in the environment and ensure better control over the quality of input streams to prevent fouling and scaling, thus improving process performances.

Luo et al. [299] investigated such strategy with coupled ED and RED stacks in continuous and batch-wise mode using NaCl and artificial sea water solutions. Concentrated brine generated by ED was used as high-salinity stream (HSS) feed for RED. Although they did not recover electric power generated in excess, they found out that optimal energy output is achieved when resistance of RED and ED stacks is comparable. Other desalination technologies have been used at small-scale to provide HSS for RED. RO coupled with membrane distillation produced superior NaCl concentration allowing the downstream RED to exhibit a power density up to 2.4 W/m² and near-zero liquid discharge desalination [301]. However, a detailed cost-efficiency analysis would be needed to establish the eco-efficiency of this strategy, especially when considering thermal energy requirements. Tristán et al. [302] carried out such evaluation for a SWRO-RED process. Their detailed LCA study demonstrated the low environmental impact of RED (comparable to solar and wind power systems) and identified that the fabrication of PES membrane spacers is the step with the highest energy cost. While functional, the SWRO-RED strategy would require optimization for better energy recovery and eco-efficiency. SWRO-MCDI-RED was investigated at lab scale and showed better performances and lower energy consumption (by 17%) when compared to two-pass RO [258]. The eco-efficiency evaluation of a multi-effect distillation (MED)-RED process recycling low-temperature waste heat showed promising results when comparing with other sustainable technologies [303].

An alternative configuration consists in implementing “upstream” RED associated with ED or other desalination technologies in order to readily supply power from RED to ED [304]. Recently, a salinity gradient energy storage system (SGESS) was investigated [305]. The two-step process combined a RED phase (discharging) producing energy from streams of different salinities, followed by an ED phase (charging) to regenerate the initial streams at higher salinity than natural streams. Cost analysis showed competitiveness for occidental energy markets if a 10-years lifetime is achieved. Life cycle assessment of a projected full-scale SGESS process led to similar as Li-ion battery productions, albeit in the first quintile of such studies in terms of eco-efficiency results.

**Figure 24:**
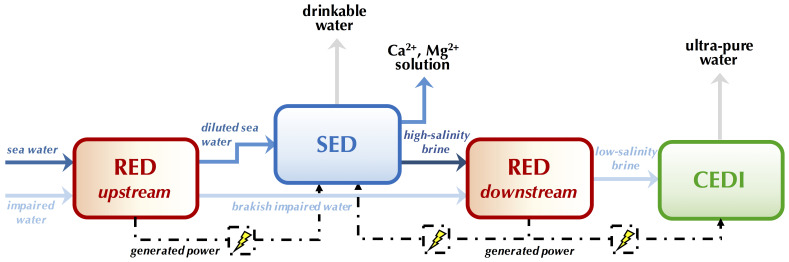
Theoretical PFED strategy (adapted from Campione et al. [10]). RED: reverse electrodialysis; SED: selectrodialysis; CEDI: continuous deionization.

Campione et al. [10] proposed a more complete RED-ED coupling strategy including two RED stacks. The “upstream” RED stack would function as pre-treatment for a more controlled salinity of the ED feed, while the “downstream” stack would act as post-treatment for energy conversion. Further purification by CEDI or recirculation of the low-salinity brine produced by the second RED equipment in the HSS input of the first RED stack would prevent any liquid effluent disposal (zero liquid discharge) providing even better sustainability. Even though current RED capabilities make PFED seems theoretical in such scenario, SED could be used in replacement of ED in order to limit the quantity of divalent cations such as Mg^2+^ in the feed of the downstream RED, thus leading to better power densities (Figure 24). The resulting Mg^2+^-enriched effluent could be valorized through struvite precipitation [166] (see Section 5.3).

### 5.2. Zero Liquid Discharge (ZLD) Strategies for Water Depollution

There are several options for managing water concentrates: direct discharge, deep well injection, discharge to a wastewater treatment facility or zero liquid discharge (ZLD), the most interesting approach from a sustainable point of view as it eliminates liquid effluent disposal [139,306]. Usually, ZLD strategies involve three steps: concentration, evaporation and crystallisation [154]. As mentioned in part Section 5.1.2, electrodialytic technologies have great potential in carrying out some of those steps as they limit more energy-consuming thermal treatments. In this part, we present recent ED strategies with potential for ZLD aiming at the maximal reuse of liquid effluents.

#### 5.2.1. Municipal Wastewaters

**Figure 25:**
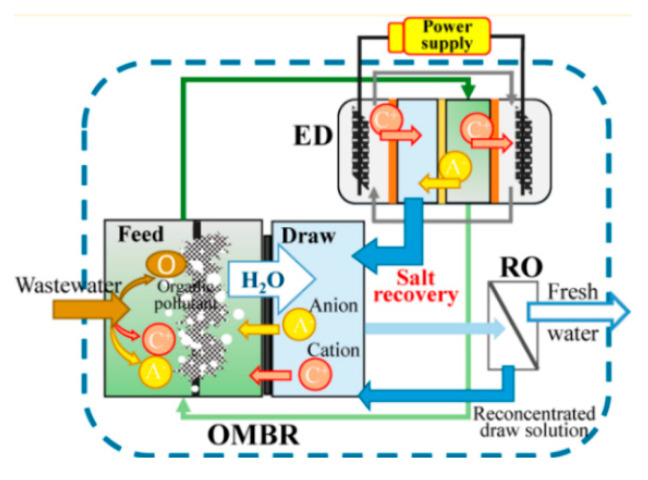
Wastewater treatment and water purification for zero liquid discharge (ZLD) by forward osmosis (FO) osmotic membrane bioreactor (OMBR)-ED (reprint with permission from Lu and He [307]. Copyright (2015) American Chemical Society). OMBR: osmotic membrane bioreactor; ED: electrodialysis; RO: reverse osmosis.

The valorization of municipal effluents in medium to low-purity grade water exhibits huge potential for agriculture as it decreases the use of potentially drinkable water such as ground and river water. An original strategy for wastewater treatment with potential for ZLD was implemented by Lu and He [307]. As depicted in Figure 25, it consists in coupling an osmotic membrane bioreactor (OMBR) with conventional ED and RO. The OMBR includes biological treatment in the feed compartment of a conventional FO in order to improve water recovery. However, salt accumulation in FO tends to induce flux reduction and scaling in such systems, even leading to reverse fluxes of soluble species [298]. The addition of an ED step to desalinate the feed mitigated this phenomenon while allowing salt concentration in the draw. Satisfactory water flux was achieved over 24 days for moderate energy consumption (1.9 kWh/m³) [307]. A similar process was tested by Zou and He [308] as part of a strategy for water reclamation and fertigation (fertilization of crops by using water-soluble nutrients in irrigation waters [298]). Using a fertilizer (diammonium phosphate) solution as the draw leads to efficient water recovery (96.6% with 0.8 kWh/m³ power consumption). Moreover, at the end of the process the low NaCl content of the fertilizer-containing draw promotes its potential at larger scale for fertigation, therefore reducing liquid effluents to be disposed. In areas prone to fresh water deficiency like Egypt, untreated drainage wastewaters have been mixed directly with the Nile river water for irrigation. While it reduces the waste of potable water, the poor water quality is detrimental to crops. Wastewater treatment using electrodialytic technologies has been investigated by Abou-Shady [309] in order to counter these deleterious practises. He proposed a global strategy for the sustainable development of the whole country, providing insights for future projects in similar areas. Vineyard et al. [310] carries out an economic analysis for nitrogen recovery in wastewaters, which could be valorize in crops through soil amendment. They found out that ED would be more eco-efficient than more mature nitrification/denitrification systems.

Modern waste water treatment plants (WWTPs) are aiming at maximal water reclamation (ideally ZLD) while keeping the lowest energy consumption. They involve several steps including UV treatment, coagulation, floculation, decantation and filtration [271]. Reclaimed water from a shoreline WWTP was successfully used in combination with 1-µm filtered UV-treated sea water by Gómez-Coma et al. [271] as feed streams for RED. Their eventual strategy consists in powering shoreline WWTPs with RED for more eco-efficient wastewater treatment. In that respect, Luque Di Salvo et al. [311] used municipal wastewater for energy production by RED and study the best strategies for long-term operation (10 days). Although it was pre-treated in a WWTP with membrane bioreactors, the RED feeding stream led to significant fouling. Membrane cleaning strategies were investigated including alkaline backwashing and pulsed electric fields (PEF) in ED mode which helped preventing fouling [311]. These results expand the applicability of RED for energy production as well as the valorization potential of municipal wastewaters.

#### 5.2.2. Industrial Wastewaters

Industrial wastewaters are more susceptible to contain hazardous pollutants compared to civil wastewaters, e.g., heavy metals, acids, bases or residual waste from oil, gas, coal recovery and energy conversion [306]. When dealing with industrial wastewaters, ED is considered as a more eco-efficient alternative than RO for ZLD as it achieves higher concentrations and lower volumes [312].

Recent ED strategy developments include a process coupling UF with ceramic membranes and ED to remediate textile wastewaters [313]. Pollution of the output stream was low (50 mg O_2_/L) as measured by chemical oxygen demand (COD) and final TDS concentration was 210 ppm for 97% recovery meeting thresholds for reuse as low-grade purity water. Pisarska et al. [314] studied the removal of sodium sulfate by electro-electrodialysis in wastewaters generated by cyclohexanone production. They managed to divide by three the COD of the initial waste and generated H_2_SO_4_ and NaOH as co-products. Although not free of organic contaminants, these acid/base solutions could be reused onsite for cleaning purposes. Similarly, a ZLD strategy involving novel selective bipolar-membrane electrodialysis (SBMED) was developed to remediate industrial wastewaters, leading to the generation of valuable products HCl, H_2_SO_4_, NaOH, purified water. Its ZLD feature and current efficiencies of 90% for NaOH and 50% for HCl make it a promising remediation process [315]. Other SED-based approaches were studied to generate acid and base using wastewaters from chemical or desalination plants which could then be resupplied in commodity chemicals. SED-EDBM processes reached a current efficiency of 70% and 1.75 kWh/kg NaOH in terms of energy consumption [316]. The monovalent-enriched and divalent enriched streams produced by SED led to HCl and H_2_SO_4_, respectively, after EDBM. High purities were reached for power consumption of 4.2–5.8 kWh/kg of product [317]. The highest NaOH and HCl purities (99.99%) were achieved by BMSED (integration of BM in the SED stack) [167]. However further investigation dealing with the eco-efficiency of the process would be required.

The treatment of hydrometallurgy effluents was investigated by means of SED-IX [318]. SED was used to produce an effluent containing arsenic in anionic form (H_2_AsO_4_^−^) and a copper/zinc-rich stream. Zn^2+^ and Cu^2+^ were then separated by two successive ion-exchange steps using solvent impregnated resins leading to 70% and 98% recovery, respectively. This strategy shows great potential as an alternative method for copper sourcing as copper mines are a major polluting industry. Tailings and other mine wastes tend to release heavy metals leading to ground water contamination. ED is currently under study as a way to remediate tailings. However, despite their recent LCA of tailings management in Norway copper mines, Song et al. [319] were unable to clearly demonstrate the feasibility of ED. A ZLD strategy coupling IX and EDBM was evaluated to remediate desulfurization wastewaters from coal-fired plants by removing major scaling contributors Ca^2+^ and Mg^2+^ [320]. The acid/base produced were suitable for resin regeneration. Polymer-flooding is used for enhanced oil recovery of oil deposit. Brackish water remains the major ingredient for such process leading to large quantities of oil/polymer/brine wastewaters. Sosa-Fernandez et al. [132] demonstrated the feasibility of electrodialytic treatment to remediate those wastewaters with low-energy costs (4.0 kWh/m³) and reuse them for polymer-flooding, thus limiting brine disposal. Improvements regarding the treatment of wastewaters generated by fossil energy industries (oil, gas, coal…) provide relevant technological insights. However, the sustainability of such applications is difficult to support due to the ecological flaws inherent to the recovery of fossil resources and their combustion for energy production.

### 5.3. ED Strategies for the Recovery of High-Purity Chemicals

Ideally the recovery of chemicals should be associated with strategies dealing with sea water desalination or wastewater depollution for better eco-efficiency, as exemplified in the previous parts. Yet, ZLD is not always attainable. In order to review relevant studies focusing on final products and their sustainable uses, this part puts the emphasis on strategies leading to high-purity products with potential for a direct utilization as high-value chemicals.

**Figure 26:**
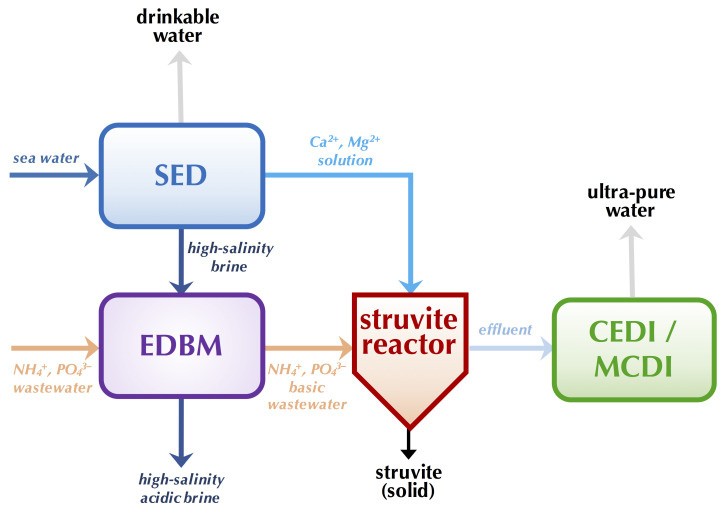
Strategy for struvite production from sea water and wastewater (adapted from Gao et al. [324]). SED: selectrodialysis, EDBM: electrodialysis with bipolar membranes; CEDI: continuous deionization; MCDI: membrane capacitive deionization.

As previously mentioned, electrodialytic technologies demonstrates suitable feasibility for the separation of nutrients used in agriculture as fertilizers. The retrieved chemicals are water-soluble supporting their potential for fertigation [298]. Potassium sulfate (K_2_SO_4_) was produced by SED [321] and EDM coupled with a NF module (EDM-NF) [145]. As a cheaper alternative to other chlorine-free potassium fertilizers, it still requires extensive processing with high energy costs to be purified. The EDM-NF strategy proposed by Trivedi et al. [145] was clearly oriented towards eco-efficiency. With current efficiencies of about 70%, they obtain K_2_SO_4_ with >99% purity at 80 g/L, although the final NF flux was half of its initial value. The low energy consumption of the EDM step (1.3 kWh/kg) is promising for eventual scale-up considerations which should include the energy evaluation of NF and additional evaporation steps. The market of struvite (MgNH_4_PO_4_·6H_2_O) as an ammonium and phosphate slow-release fertilizer has been increasing for the past decades. Researchers developed strategies to ensure low cost and environment-friendly production [156]. Although sea water can be readily used for struvite production by precipitation due to its high magnesium content, it would generate large amount of wastewater. To solve this issue, Ghyselbrecht et al. [166] proposed to selectively concentrate magnesium from North Sea water by SED before struvite production. Figure 26 display a possible process for struvite precipitation from marine Mg^2+^. While the authors managed to double Mg^2+^ concentration in real sea water, an increase in Ca^2+^ was also observed which could impair the efficiency of struvite precipitation [166]. Other strategies involve the cyclic combination of ED and SED with a struvite reactor for phosphate and ammonium recovery (up to 100% and 94%, respectively) [164,322]. Phosphate recovery with integrated SED/crystallization process was lower while it led to a 44% purity [163]. Focusing on nitrogen (NH_4_^+^) recovery form wastewaters, Ward et al. [323] implemented ED at pilot-scale and used struvite crystallization as a pre-treatment. The concentration of the final product was eight-times higher than in the initial feed. The current efficiency of 76% and energy consumption of 4.9 kWh/kg N obtained at pilot-scale demonstrated the economic feasibility and potential eco-efficiency of the process. More recently, Gao et al. [324] investigated struvite precipitation by an integrated EDBM-MCDI process. EDBM efficiently increased the pH of the ammonium and phosphate-enriched stream to enhance precipitation with MgCl_2_ in the struvite reactor. Figure 26 integrates this strategy for struvite precipitation with Mg from sea water. While 100% PO_4_^3−^ was recovered as solid struvite, the excess NH_4_^+^ contained in the resulting effluent was further concentrated by MCDI leading to a 77% global recovery and low energy consumption (3.2 kWh/kg N).

The segregation of nitrogen and phosphorus nutrients has also been studied as part of volatile fatty acids (VFAs) recovery. VFAs such as acetic acid are valuable chemicals that can be used as monomers in biopolymer synthesis and other high-value added applications. They can be sustainably produced from biomass using mixed culture bioreactor. A strategy was devised for their recovery whilst retaining nutrients to support continuous fermentation in the bioreactor [325]. The extraction of VFAs relied on a polytetrafluoroethylene (PTFE) membrane stack while their continuous concentration was achieved with ED. Nutrients in the form of PO_4_^3−^, NO_3_^3−^ and NH_4_^+^ were successfully retained in the dilute stream, and 98% of acetic acid was recovered in the concentrate.

High-purity inorganic salts have been produced by efficient systems combining membrane and electromembrane technologies. An integrated NF-ED process named “Fracsis” was investigated for the production of Ca^2+^ and SO_4_^2−^ and led to 98% and 96.6% rejections, respectively, at pH 6.5 for which no scaling was observed [326]. The higher water recovery (77%) compared to RO and the moderate energy requirements (8 kWh/m³) demonstrates the potential of “Fracsis” for the production of high-purity salts and the reclamation of fresh water.

Lithium is not used exclusively for the production of Li-ion batteries, but also involved in CO_2_ purification and nuclear energy production among others [191,225]. As ionic lithium is naturally occurring in salt lakes along with other cations such as Mg^2+^, extraction technologies have been developed, among which ED exhibit the lowest energy consumption [168]. Jiang et al. [191] implemented an ED-EEDBM in order to produce high-purity LiOH. Conventional ED helped concentrate Li_2_CO_3_ (4 times), while EEDBM yielded LiOH with 95% purity. The high current efficiency achieved (99%) and moderate energy consumption (8 kWh/kg) led to better performances than ED alone. Using a similar strategy, Qiu et al. [168] produced LiOH by SED-BMSED. Using monovalent permselective IEMs led to an improved selectivity for Li^+^ vs. other cations Mg^2+^ and Ca^2+^, both during pre-concentration by SED and LiOH production with BMSED. Even though current densities were lower than for the previous process (41% SED, 58% BMSED), energy consumption was still moderate (3.8 kWh/kg SED, 5.5 kWh/kg BMSED) and the final purity higher with close to 99%. It demonstrates the potential of the process as an eco-efficient strategy for lithium production.

### 5.4. Eco-Efficient Production of Food and Nutraceuticals with ED

ED technologies are particularly suitable for the low-margin high-volume food industry. They allow a significant increase in the value of food products at low cost. Already implemented in pilot plants for several applications including depollution and production of organic acids, EDBM shows great potential for new avenues in food processing [128,327]. Moreover, the emergence of EDFM (see Section Section 4.10) this past decade, is providing specific separation applications leading to high-added value products.

#### 5.4.1. Fruit Juice

Deacidification of cranberry juice by electrodialytic processes was extensively studied as a green strategy to improve its health benefits. The reduction of its organic acid content combined with its high polyphenol content gives treated cranberry juice potential as a nutraceutical. Furthermore, it was demonstrated that removal of cranberry juice protects against disruption of in vitro intestinal cell barrier integrity which is mainly due to citric acid [328], and that a minimal deacidification rate of 37% was necessary to reach this level of protection [329]. From the several approaches tested (EDBM, EDUF, EDBMUF), EDBM with AEMs yielded the best performance [329]. The eco-efficiency of the strategy was evaluated at a semi-pilot scale by Faucher et al. [330]. The process involved the recycling of the recovery solution generated during electro-deacidification of the juice. It led to reduce energy consumption almost by half without any impact on the product quality. To further improve the eco-efficiency of the process, PEF could be applied as they showed positive impact on organic acid removal by decreasing concentration polarization, thus increasing efficiency [122]. Life cycle assessments of the production of deacidified cranberry juice were carried out [284,331]. Extracted cranberries were identified as the main co-product of the process due to their potential high added-value after several drying steps. Interestingly, the authors also brought to light pomace as another co-product due to its interest as a fertilizer. Organic acids could be recovery for use as preservative in food-related applications. Compared to non-deacidified juice and juice processed using IX, cranberry juice was more eco-efficiently produced by EDBM when proper scenarios were considered [284]. This process was also found slightly more eco-efficient than production by salt precipitation, especially since this last process had a negative impact on the quality of the product and prevented organic acid recovery [331]. These results are of great significance for the field as they support the relevance of ED technologies towards sustainable practises.

#### 5.4.2. Dairy Products

**Figure 27:**
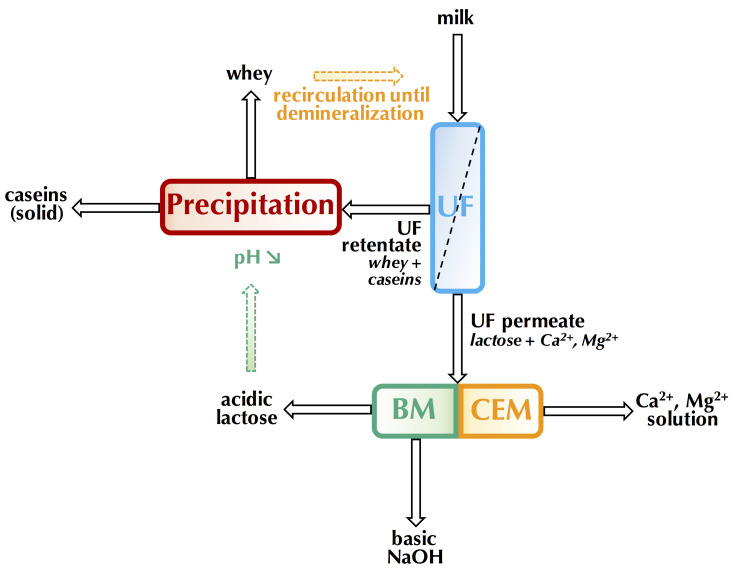
Eco-efficient milk processing by EDBM-UF for casein production (adapted from Mikhaylin et al. [335]). UF: ultrafiltration; BM: bipolar membrane; CEM: cation-exchange membrane.

The research of sustainable ways for the production of bioactive peptides is a major challenge for the food industry. Although these protein-derived compounds exhibit remarkable biological properties, they can be sensitive to external factors (temperature, light, oxidation…). An electro-membrane process is thus a suitable option for their separation [332]. Mikhaylin et al. [88] recently proposed a novel approach for the recovery of caseins from milk as sources of bioactive peptides. Their EDBM-UF process allowed continuous operation as illustrated in Figure 27. Caseins were kept in the UF retentate while the lactose-enriched permeate was used as feed for EDBM, thus avoiding fouling in the electrodialytic cell. Caseins were precipitated as curds due to the decreased pH induced by H^+^ generation by EDBM. In the same time, the generation of OH^−^ on the other side of the bipolar membrane and the migration of Na^+^ from the electrolyte produced NaOH as a co-product that could be used for cleaning. Interestingly, demineralized whey was also produced in the UF retentate after filtration of the caseins. This was due to the recirculation of the lactose-enriched permeate in the EDBM cell and its demineralization through the CEM, which produced an additional fraction enriched in divalent cations. In addition, it was demonstrated that the caseins and caseinates produced by EDBM were equivalent in terms of lactose and protein contents to their respective chemically produced caseins and caseinates, but presenting lower sodium content and higher magnesium and calcium contents [137]. The higher calcium content is very interesting since this high concentration induced by the new EDBM process add an increased value to the final product. Indeed, the high calcium content of the produced caseins and caseinates could justify their use as a calcium rich protein source, notably in osteoporosis patient, since calcium is essential to bone health [333]. Furthermore, the fact that the calcium is likely bounded to milk protein makes it somewhat pH resistant and ensured its absorbability [334]. This could make these caseins or caseinates from the new EDBM process suitable as improved protein-based calcium supplement both for its enhanced nutritionals quality and for the fact that it is produced by a green process [137]. The LCA of the process demonstrated a 10% reduction of the environmental impact when compared to the usual chemical method for precipitation. Casein yield was also increased by 8% and additional value-added co-products were generated leading to a more optimal valorization [335].

As previously mentioned, whey is a by-product from the dairy industry generated in large amounts. Its disposal raises environmental issues due to its high organic acid and saline content [113]. Chen et al. [336] applied EDBM to salty whey to generate high-purity HCl and NaOH as cleaning chemicals. Their simple strategy to recycle 9% of the produced base as a salt precipitation pre-treatment improved performance (3.0–3.6 mol/L purity and 6.6 kWh/kg energy consumption) and led to a 99% demineralization of the whey effluent which could then be valorized as a source of bioactive peptides.

#### 5.4.3. Meat and Fish Industries

Commercial ingredients derived from food waste are of limited occurrence when excluding dairy by-products. However, recent works have investigated by-products from fish and meat processing as a source of high-value molecules [332]. Adopting an eco-efficiency approach similar to biorefinery strategies, researchers demonstrated that ED technologies, especially EDUF could be integrated in those industries to support a circular food economy and improve the eco-efficiency of the all production line. Indeed, the circular economy is an economy that operates in a loop, thus avoiding waste. Its objective is to produce goods while strongly limiting the consumption of raw materials and non-renewable energy sources.

Przybylski et al. [337] worked on an eco-efficient strategy to recycle blood generated during meat processing into meat preservatives exhibiting antimicrobial activity. While plasma, the colorless fraction is valorized, cruor, the remaining 40% is often discarded. The authors produced a cruor peptic hydrolysate for treatment by EDUF in order to recover cationic antimicrobial peptides. The fraction was obtained at the lowest energy consumption (3.4–5 kWh/kg) when using 5–8% hemoglobin in the initial feed. The good antimicrobial results led them to optimize the process by studying the influence of the degree of hydrolysis (DH) [338] and the pH [339]. By targeting TSKYR (α 137–141, 653 Da), an antimicrobial peptide with interesting properties for meat taste preservation, they found that pH 9 increased the purity of the fraction, while a 5% DH allowed a 13-fold concentration of TSKYR compared to the initial hydrolysate. In keeping with the concept of circular economy, the blood waste from slaughterhouses can thus be recycled into raw materials of the meat industry for production of bioproducts with improved functions. Several studies are dealing with similar processes for the valorization of fish by-products [236,238,239,340]. In general, a multi-step strategy is implemented: recovery of the by-product (including deboning, homogenization), preparation of the hydrolysate (enzymatic hydrolysis), selective separation into bioactive fractions by EDUF, concentration and drying for preservation. Salmon and rainbow trout frames produced peptide fractions exhibiting antidiabetic [236] and also antihypertensive activities [340]. Due to the intrinsic nature of these by-products, fouling and long-term maintenance of the membranes remains a challenge. However, the future developments in EDUF-PEF are expected to improve membrane lifespan to reduce costs and limit fouling for better performances [105].

## 6. Conclusions and Perspectives

Electrodialytic technologies are everywhere: water purification, energy production, depollution, agrochemical, biotechnology, food industries, etc. However, it appears that due to the new advances in sciences, some well-known ED technologies are regaining great amounts of interest in the present economical context and ecological crisis. Indeed, the new knowledge on pulsed electric fields, electroconvective vortices, overlimiting conditions and reversal mode as well as their recent demonstrations of applications is boosting this renewed interest for some of these old ED processes. However, hurdles are still high when dealing with scale-ups and real-life conditions. Pulsed electric fields, electroconvective vortices, salinity gradient and selectivity of membranes will still be the main focuses for future studies to improve their implementation in the industry. Furthermore, looking at the current research trends, potable water and wastewater treatment to answer the world population demand as well as the production of value-added bioactive products in a circular economy due to the health concerns and the growing aging population will probably be the main applications to be developed and improved.

The awareness that those technologies or derivate ones are attractive to meet the new challenges of eco-efficiency and/or circular economy also led to develop innovative sustainable strategies integrating ED processes. Amongst others, the combination of various technologies with electrodialytic processes at different steps of the production and the maximization of high added-value co-products seems like a viable strategy according to recent life cycle assessment studies. The possibility to recycle membranes for other types of applications also holds great potential. In opposition to large-scale developments, small-scale prototypes and portable devices should be encouraged for local or emergency supply. This would lead to concrete solutions to provide pure water access to remote and poorer areas as they are those who need it the most. However, to prove the eco-efficiency or sustainability of such processes or process strategies, more life cycle assessments will be necessary to convince people of the merits or validity of these technologies and their couplings.

## Figures and Tables

**Table 1:** Mitigation of different fouling natures during electrodialysis, of model and complex solutions, under pulsed electric field regimes and impact on energy consumption and mass transfer (demineralisation rate, removal efficiency, current efficiency or molecule recovery). DR: demineralization rate, CC: continuous current, CE: Current efficiency, LARR: Lactic acid recovery rate, RE: Removal efficiency, REC: Relative energy consumption.

Fouling	Solution	Current Density	Pulse/Pause	Pulse/Pause Ratio (Frequency)	Membrane Studied	Fouling Mitigation	Energy/Relative Energy Consumption	Mass Tranfer Efficiency	References
**Amino acid/Peptide**	Snow crab by-product hydrolysate	20 and 40 V	2 s/0.2 s vs. CC	10 (0.45 Hz)	CEM and AEM	- Significant fouling reduction by PEF - AEM fouling depends strongly on applied voltage but not for CEMs.	CC: 806 Wh/g of peptide recovered 20 V- PEF: 656 Wh/g 40 V-PEF: 894 Wh/g	The peptide/amino acid migration was the same between CC and PEf but was increased by 30% at 40 V DR slightly higher in PEF mode at 40 V.	[105]
**Humate**	Model salt solution (0.01 M NaCl + 0.01% sodium humate)	6 mA/cm^2^	-	60, 100 and 200 Hz	AEM	- 100 Hz gave the least fouling tendency	Not indicated	Not indicated	[39]
			-	10-500 Hz	CEM and AEM	- 30 Hz presented the least fouling	Not indicated	CE ≈ 84% at 30 Hz CE ≈ 77% for CC	[102]
**Lignin**	Kraft black liquor	330 A/m^2^	6 s/6 s 6 s/12 s 6 s/18 s 6 s/24 s	1 (0.08 Hz) 0.5 (0.055 Hz) 0.33 (0.041 Hz) 0.25 (0.033 Hz)	CEM and BPM	- No fouling of BPM and CEM for 6 s/24 s	3.2 Wh/g NaOH	CE ≈ 57%	[106]
						- Increase in current efficiency with an increase in pause duration	2.9 Wh/g NaOH 2.7 Wh/g NaOH 2.6 Wh/g NaOH CC = 3.6 Wh/g NaOH	CE ≈ 60% CE ≈ 70% CE ≈ 80% CE ≈ 48%	
**Natural organic matter**	Ion-exchange spent brine	34 mA/cm^2^	2 s/0.5 s vs. CC	4 (0.4 Hz))	AEM and CEM	- Reduction in deposition of negatively charged NOM fractions	No difference in REC whatever the condition, REC ≤ 2.1 Wh/g NaCl produced	DR = 89–96% DR =90–91% for CC	[107]
	Sodium gluconate mother liquor	Between 9 and 15 mA/cm^2^	4 s/1 s vs. CC	4 (0.2 Hz)	CEM and AEM	- 70% Decrease in AEM fouling vs. Constant voltage	194.4 kWh/t	DR = 25.2%, CE = 45.9%	[115]
						- No fouling for CEM whatever the mode of voltage	201.7 kWh/t for CC	DR = 18.9%, CE = 44.6% for CC	
	Lysine fermentation broth	10 mA/cm^2^	Half-wave power (37 min constant current over 180 min) and CC (12min over 180 min)	Frequency of 60 Hz for half-wave power	CEM and AEM	- 22% decrease in final stack resistance	REC = 2.7 kWh/m^3^ for half-wave REC = 3.8 kWh/m^3^	DR = 45.6% DR = 34.1% for CC	[116]
	Lysine fermentation waste	25 mA/cm^2^	Half-wave power (105 min constant current over 300 min) and DC power (125 min over 420 min)	-	CEM and AEM	- 65% decrease of the electrodialysis membrane fouling index for half-wave power	REC = 58 kWh/m^3^ for half-wave REC = 124 kWh/m^3^	RE = 66.3 and 85.2% for ammonium and sulfate respectively with half-wave. RE = 52.2 and 65.4% with CC	[117]
**Protein**	Salt solution containing casein	10, 20 and 30 mA/cm^2^	10 s/10 s 10 s/40 s	1 (0.05 Hz), 0.25 (0.02 Hz)	AEM	- No fouling at 10 s/40 s whatever the current density - No fouling at 10 s/10 s and 10 mA/cm^2^	0.25, 1.09 and 2.85 Wh for 10 s /10 s 0.25, 1.01 and 2.75 Wh for 10 s /40 s 0.30, 1.33 and 2.77 Wh for CC	DR = 10.5, 13.1 and 18.7% at 10, 20 and 30 mA/cm^2^ whatever the current mode.	[103]
**Protein fouling and scaling**	Salt solution containing whey protein	15 mA/cm^2^	10 s/40 s vs. CC	0.25 (0.02 Hz)	CEM and AEM	- Decrease in mineral fouling (Ca and Mg by 16 and 24%) in basic conditions - 18% Decrease in protein fouling in acidic condition	5.81 Wh for PEF conditions 5.87 Wh for CC	DR = 74.1, 64.7 and 79.5% in acid, basic and acid/base separated conditions DR = 56.3, 54.8 and 58.8% for CC	[104]
**Scaling**	Model salt solution (Mg/Ca = 2/5)	40 mA/cm^2^	10 s/10 s 10 s/33.3 s	1 (0.05 Hz) 0.3 (0.023 Hz)	CEM and AEM	- 10 s/10 s protects the AEM during three consecutive runs	14.7 Wh	DR= 68.4%	[110,111,114]
						- Delayed fouling growth at 10 s/33.3 s on CEM during three consecutive runs	22.1 Wh 20.2 Wh for CC	DR = 68.1% DR = 61.8% for CC	
			5 s/10 s 10 s/20 s 5 s/5 s 10 s/10 s 10 s/5 s 20 s/10 s	0.5 (0.066 Hz) 0.5 (0.033 Hz) 1 (0.1 Hz) 1 (0.05 Hz) 2 (0.066 Hz) 2 (0.033 Hz)	CEM and AEM	- 10 s/5 s and 5 s/5 s suppressed CEM fouling on the diluate side - 10 s/5 s reduced fouling also on concentrate side - No severe scaling on AEM	12.4 Wh 11.4 Wh 14.0 Wh 13.4 Wh 14.6 Wh 14.4 Wh	DR = 50.6% DR = 48.9% DR = 59.6% DR = 51.6% DR = 58.5% DR = 57.3%	[111]
			1 s/0.33 s 1 s/1 s 2 s/0.5 s 2 s/0.67 s 3 s/0.3 s 3 s/1 s	3 (0.75 Hz) 1 (0.5 Hz) 4 (0.4 Hz) 3 (0.37 Hz) 10 (0.303 Hz) 3 (0.25 Hz)	CEM and AEM	- 2 s/0.5 s suppressed scaling on both sides of CEM - 2 s/0.67 s suppressed scaling only on the CEM concentrate side - Absence of scaling on AEM	15.1 Wh 14.0 Wh 17.8 Wh 17.3 Wh 17.3 Wh 16.2 Wh	DR = 72.5% DR = 71.6% DR = 79.3% DR = 78.8% DR = 74.8% DR = 75.5%	[112]
			2 s/0.5 s 2 s/0.67 s	4 (0.4 Hz) 3 (0.374 Hz)	CEM	- Membrane less scaled at 2 s/0.5 s	11.8 Wh 12.8 Wh	DR = 59.0% DR = 57.2%	[66]
	Milk	20 mA/cm^2^	2 s/0.5 s vs. CC	4 (0.4 Hz)	CEM and AEM	- Inhibition of scaling formation - Inhibition of OH^−^ leakage	Not indicated	Not indicated	[88]
	Model salt solution (Mg/Ca = 2/5)	10.5 mA/cm^2^	15 min/15 min 15 min/7.5 min 5 min/5 min 1 min/1 min	1 (5.5 10^-4^ Hz) 2 (7.4 10^-4^ Hz) 1 (1.6 10^-3^ Hz) 1 (8.3 10^-3^ Hz)	CEM and AEM	- Scaling Reduction for 5 min/5 min and 1 min/1 min	Not indicated	Not indicated	[108]
	Sweet whey (6.5% total solids)	8.0 mA/cm^2^	0.1 s/0.1 s 1 s/0.1 s 1 s/1 s 10 s/0.1 s 10 s/1 s 10 s/10 s 100 s/1 s	1 (5 Hz) 10 (0.9 Hz) 1 (0.5 Hz) 100 (0.099 Hz) 10 (0.090 Hz) 1 (0.05 Hz) 100 (0.1 Hz)	CEM and AEM	- No visual fouling or scaling on CEMs and AEMs, except for 0.1 s/0.1 s	3591 Wh for 42% DR	79.6% DR	[64]
						- For other conditions, the limiting current density was not reached - No significant difference of membrane thickness and conductivity whatever the conditions used.	4160 Wh for 42% DR 4118 Wh for 42% DR 4161 Wh for 42% DR 4270 Wh for 42% DR 4171 Wh for 42% DR 4213 Wh for 42% DR CC = 4266 Wh for 42% DR	46.6% DR 48.5% DR 44.6% DR 42.8% DR 43.8% DR 43.0% DR CC = 43.9% DR	
	Acid whey	10 mA/cm^2^	25 s/25 s 50 s/10 s	1 (0.02 Hz) 5 (0.016 Hz)	CEM and AEM	- 25 s/25 s pulse/pause decreased drastically scaling bycalcium phosphate	6.2 Wh/g lactic acid	44.4% LARR, 67.1% DR	[113]
							7.9 Wh/g lactic acid CC = 9.3 Wh/g lactic acid	41.6% LARR, 64.2% DR CC = 37.2% LARR and 64.0% DR	
			5 s/5 s 15 s/15 s 15 s/25 s 25 s/25 s 25 s/15 s 25 s/35 s 35 s/35 s 35 s/25 s	1 (0.01 Hz) 1 (0.033 Hz) 0.6 (0.025 Hz) 1 (0.02 Hz) 1.66 (0.025 Hz) 0.71 (0.016 Hz) 1 (0.025 Hz) 1.4 (0.016 Hz)	CEM and AEM	Scaling was greatly mitigated at: - conditions with high frequency (5 s/5 s and 15 s/15 s) - conditions with low pulse/pause ratios (15 s/25 s and 25 s/35 s)	5.6 Wh/g lactic acid 5.3 Wh/g lactic acid 5.6 Wh/g lactic acid 5.8 Wh/g lactic acid 6.2 Wh/g lactic acid 6.2 Wh/g lactic acid 6.7 Wh/g lactic acid 6.7 Wh/g lactic acid	Global DR (including calcium, magnesium, sodium, potassium, and phosphorus) of 67.3% and LARR of 44.5% whatever the PEF conditions used.	[83]
**Scaling and partially hydrolyzed polyacrylamide (HPAM)**	Brackish water, Brackish water + polymer and Brackish water + polymer +oil	3.2 mA/cm^2^	0.1 s/0.1 s 1 s/1 s 1 s/3 s 3 s/1 s 10 s/10 s 10 s/30 s 100 s/100 s 100 s/300 s	1 (5 Hz) 1 (0.5 Hz) 0.33 (0.25 Hz) 3 (0.25 Hz) 1 (0.05 Hz) 0.33 (0.025 Hz) 1 (5 10^−3^ Hz) 0.33 (2.5 10^−3^ Hz)	CEM and AEM	- Less organic fouling during pulsed regimes of 10 s/30 s and 1 s/3 s - However, more minerals (Na, Ca, O, and S) were spotted on the concentrate side of the AEM.	≈ 0.52 kWh/m^3^ ≈ 0.57 kWh/m^3^ ≈ 0.50 kWh/m^3^ ≈ 0.74 kWh/m^3^ ≈ 0.64 kWh/m^3^ ≈ 0.54 kWh/m^3^ ≈ 0.79 kWh/m^3^ ≈ 0.69 kWh/m^3^ ≈ 0.75 kWh/m^3^ for CC	DR ≈ 85.6% DR ≈ 91.5% DR ≈ 89.1% DR ≈ 87.2% DR ≈ 89.3% DR ≈ 88.4% DR ≈ 86.1% DR ≈ 85.7% DR ≈ 84.5% for CC	[84]

## Abbreviations

AEM	Anion-Exchange Membrane
BMSED	Selectrodialysis with Bipolar Membrane
CDI	Continuous Current
CEDI	Capacitive Deionization
CEM	Continuous Electrodeionization
COD	Cation-Exchange Membrane
CP	Concentration Polarization
DBL	Diffusion Boundary Layers
DC	Direct Current
DH	Degree of Hydrolysis
ECV	Electroconvective Vortex
ED	Electrodialysis
EDBM, BMED	Electrodialysis with Bipolar Membrane
EDBMUF	Electrodialysis with Bipolar Membrane and Ultrafiltration Membrane
EDFM	Electrodialysis with Filtration Membrane
EDI	Electrodeionization
EDM	Electrodialysis Metathesis
EDNF	Electrodialysis with Nanofiltration Membrane
EDR	Electrodialysis Reversal
EDUF	Electrodialysis with Ultrafiltration Membrane
EED	Electro-Electrodialysis
FCDI	Flow-electrode Capacitive Deionization
FEDI	Fractional Electrodeionization
FO	Forward Osmosis
HI	Hydrogen Iodide
HSS	High-Salinity Stream
IEM	Ion-Exchange Membrane
IX	Ion-exchange
LCA	Life Cycle Assessment
LCD	Limiting Current Density
LSS	Low-Salinity Stream
MCDI	Membrane Capacitive Deionization
MEA	Membrane-Electrode Assembly
MVA	Monovalent permselective Anion-exchange
MVC	Monovalent permselective Cation-exchange
MWCO	Molecular Weight Cut-Off
NF	Nanofiltration
NOM	Natural Organic Matter
OMBR	Osmotic Membrane Bioreactor
pEDR	Electrodialysis Reversal under Pulsed Electric Filed
PEF	Pulsed Electric Field
PFED	Power-Free Electrodialysis
PIM	Polymer Inclusion Membrane
PIM-ED	Electrodialysis with Polymer Inclusion Membrane
REC	Relative Energy Consumption
RED	Reverse Electrodialysis
REED	Reverse Electro-Electrodialysis
RO	Reverse Osmosis
SCR	Space-Charge Region
SED	Selectrodialysis
SGESS	Salinity Gradient Energy Storage System
SGP	Salinity Gradient Power
Shock ED	Shock Electrodialysis
SLM	Supported Liquid Membrane
SW	Sea Water
TDS	Total Dissolved Solids
TFC	Thin-Film Composite
UF	Ultrafiltration
UFM	Ultrafiltration Membrane
VFA	Volatile Fatty Acid
WWTP	Waste Water Treatment Plants
ZLD	Zero Liquid Discharge
